# Comprehensive prognostic and immune analysis of a glycosylation related risk model in pancreatic cancer

**DOI:** 10.1186/s12885-023-11725-1

**Published:** 2023-12-14

**Authors:** XueAng Liu, Jian Shi, Lei Tian, Bin Xiao, Kai Zhang, Yan Zhu, YuFeng Zhang, KuiRong Jiang, Yi Zhu, Hao Yuan

**Affiliations:** 1 Pancreas Center, The First Affiliated Hospital of Nanjing Medical University, Nanjing, China; 2 Pancreas Institute of Nanjing Medical University, Nanjing, China; 3 Department of General Surgery, The First Affiliated Hospital of Nanjing Medical University, Nanjing, China; 4 Department of Pathology, The First Affiliated Hospital of Nanjing Medical University, Nanjing, China; 5 NHC Key Laboratory of Antibody Technique, Nanjing Medical University, Nanjing, China

**Keywords:** Pancreatic cancer, Cancer glycosylation, Prognostic model, Bioinformatics, B3GNT8

## Abstract

**Background:**

Pancreatic cancer (PC) is a malignant tumor with extremely poor prognosis, exhibiting resistance to chemotherapy and immunotherapy. Nowadays, it is ranked as the third leading cause of cancer-related mortality. Glycation is a common epigenetic modification that occurs during the tumor transformation. Many studies have demonstrated a strong correlation between glycation modification and tumor progression. However, the expression status of glycosylation-related genes (GRGs) in PC and their potential roles in PC microenvironment have not been extensively investigated.

**Method:**

We systematically integrated RNA sequencing data and clinicopathological parameters of PC patients from TCGA and GTEx databases. A GRGs risk model based on glycosylation related genes was constructed and validated in 60 patients from Pancreatic biobank via RT-PCR. R packages were used to analyze the relationships between GRGs risk scores and overall survival (OS), tumor microenvironment, immune checkpoint, chemotherapy drug sensitivity and tumor mutational load in PC patients. Panoramic analysis was performed on PC tissues. The function of B3GNT8 in PC was detected via in vitro experiments.

**Results:**

In this study, we found close correlations between GRGs risk model and PC patients’ overall survival and tumor microenvironment. Multifaceted predictions demonstrated the low-risk cohort exhibits superior OS compared to high-risk counterparts. Meanwhile, the low-risk group was characterized by high immune infiltration and may be more sensitive to immunotherapy or chemotherapy. Panoramic analysis was further confirmed a significant relationship between the GRGs risk score and both the distribution of PC tumor cells as well as CD8 + T cell infiltration. In addition, we also identified a unique glycosylation gene B3GNT8, which could suppress PC progression in vitro and in vivo.

**Conclusion:**

We established a GRGs risk model, which could predict prognosis and immune infiltration in PC patients. This risk model may provide a new tool for PC precision treatment.

**Supplementary Information:**

The online version contains supplementary material available at 10.1186/s12885-023-11725-1.

## Introduction

Pancreatic cancer is one of the highly malignancies of the digestive tract [1]. Due to the lack of effective prediction and treatment strategies, the 5-year OS rate of PC is currently only 12% [2]. Therefore, it is imperative to develop new prognostic assessment methods for PC. Glycosylation is a post-translational modification of proteins that serves as a crucial regulatory in controlling various physiopathological processes [3]. Multiple types of glycoconjugates disrupt crucial cancer cell processes and the tumor microenvironment, thereby promoting cancer progression [4, 5]. So far, numerous studies have demonstrated the close correlation between glycosylation and tumorigenesis [6]. Tumor cells often exhibit aberrant glycosylation alterations compared to non-malignant cells. Aberrantly expressed GRGs have also been demonstrated as biomarkers in a variety of tumors [4]. The mechanisms of PC have been deeply studied, and aberrant glycosylation alterations play an important role in PC progression, signaling, adhesion, immune response and drug resistance [7]. However, previous studies have primarily focused on the impact of individual glycosylation modification or single glycosylation enzymes on tumors [8]. The expression status of numerous GRGs in PC and their correlation with the PC microenvironment remain to be elucidated.

In this study, we conducted a comprehensive analysis of the GRGs expression status in PC by integrating RNA sequencing data and clinicopathological information from 178 PC samples and 169 normal control samples obtained from TCGA and GTEx databases. Based on the correlation between GRGs expression and patient outcomes, a 5 gene-prognostic risk model was established. In order to verify the feasibility of the GRGs risk model, sixty PC patients with detailed follow-up information were selected for external validation. Meanwhile, the relationships between the risk model and immune cell type fractions, immune checkpoints, chemotherapy drug sensitivity and tumor mutational load were further evaluated to explore underlying value of the GRGs risk model. To further investigate and validate the correlation between risk score and PC tumor microenvironment, we performed a panoramic analysis using multicolor immunofluorescence technology. Our findings indicate a significant association between GRGs risk score and both the distribution of PC tumor cells as well as CD8 + T cell infiltration. In addition, we selected a notable model gene B3GNT8, which is highly expressed but negatively correlated with prognosis. In vitro functional experiments were conducted to further demonstrate the correlation between B3GNT8 and the progression of PC.

In general, we developed a GRGs risk model, and the prognostic role of the model was identified by multifaceted analysis. The relationship between the risk score and PC tumor microenvironment were further detected and verified via bioinformatic analysis and mIHC technology. The risk model is anticipated to provide a novel method for PC personalized treatment, which may help PC patients to find precision antitumor therapy and improve the prognosis.

## Method

### Sources of gene expression and clinical data

From The Cancer Genome Atlas (TCGA, https://portal.gdc.cancer.gov/), data of RNA sequence and relevant clinical parameters (e.g. sex, age, grade, TNM stage, survival) were obtained for 179 PC patients and 4 normal samples [9]. Information on additional 165 normal samples was obtained from Genotype-Tissue Expression (GTEx, https://www.gtexportal.org/home/index.html) [10]. Glycosylation-related genes were downloaded from Glyco Gene Database (GGDB, https://acgg.asia/ggdb2/) [11]. All these data were extracted on April 17, 2022.

### Identification of differentially expressed GRGs (DE-GRGs)

The DE-GRGs between PC and normal tissues were screened with *P* < 0.05 and |log fold change|> 1 via package “limma”. Gene ontology (GO) and Kyoto Encyclopedia of Genes and Genomes (KEGG) pathway analyses were completed on the R package “clusterProfiler”. Next, we calculated the associations among the DE-GRGs using Pearson's test and displayed with the R package “corrplot”.

### Prognostic model based on identification of prognostic genes

In univariate Cox analysis, DE-GRGs associated with the survival of PC patients were screened. Then the consistency index (C-index) was used to verify the model accuracy. For further screening, OS-related GRGs were incorporated into the least absolute shrinkage and selection operator (LASSO) analysis. The independent risk GRGs associated with prognosis were further determined via multivariate cox analysis. We established a prognostic gene risk model via linearly combining the cox regression coefficient multiplied by gene expression: risk score = gene1 expression * β1 + gene2 expression * β2 + … + gene n expression * βn. Next, PC patients were categorized by the median risk score into high- and low- risk groups. To evaluate the survival of both groups, we used the R package “survival” to plot Kaplan–Meier curves. Based on receiver’s operating characteristic curves (ROC curves) and area under the curve over time (AUC), a five-year survival prediction experiment was conducted to evaluate the forecasting behaviors of clinical and genetic factors.

### Determination of independent prognostic parameters

We evaluated the applicability of the above model using multiple indicators including risk score, gender, age, grade, stage, and tumor node metastasis (TNM) classification involved in uni/multivariate cox regression to define individual risk factors. An ROC analysis was also conducted to test modeling sensitivity and effectiveness.

### Establishment of the nomogram

Based on prognostically relevant clinicopathological factors, a nomogram was built on R packages “rms” and “survival” to visualize the cox regression model. In the validation process, we calibrated the column line plots using calibration plots.

### Tumor mutational burden (TMB) and risk score

R package "survival” was used to plot Kaplan–Meier curves, aiming to assess the impact of TMB on patient prognosis. Then TMB data were combined with risk scores to evaluate the combined effect of the two on prognosis. Using the R package “maftools” to analyze the TCGA somatic variant data and visualized the top 10 mutated genes.

### Immune cell infiltration analysis

The infiltration differences in the 22 immune-related cell types between both groups were compared on R package “cibersort”. Patients were scored with TIDE (http://tide.dfci.harvard.edu). The module can compare the custom biomarker with other reported biomarkers in their forecasting response results and overall survival. Next, the relationship between common immune checkpoints (PDCD1, CTLA4, PD-L1) and risk score was analyzed via Pearson's test.

### Clinical samples with clinical information parameters

Surgically resected PC tissues and paired adjacent non-tumor tissues with confirmed by two pathologists (*Yan Zhu & Yi Zhu*) were obtained from 60 patients at Pancreatic biobank (the First Affiliated Hospital with Nanjing Medical University, China), which has passed the ISO90012015 quality certification. None of these patients received chemotherapy or radiotherapy before surgery, all of whom underwent pancreaticoduodenectomy between 2019 and 2022, and were pathologically confirmed as pancreatic cancer. OS was defined as the time between surgery and death or the last follow-up date. Approval was provided by the hospital Ethics Committee (No. 2020-SRFA-364) and written informed consents were offered by the patients.

### Cell culture

Human PC cell lines (CFPAC-1, MIA PaCa-2), mouse pancreatic cancer cell line (Pan02) and a normal human pancreatic ductal cell line (HPNE) from Shanghai Cell Bank (China) were cultured in Dulbecco’s modified Eagle’s medium (DMEM) (Bio-Channel, Nanjing, China) containing 10% fetal bovine serum (Bio-Channel) at 37℃ in a 5% CO_2_ incubator.

### Extraction of RNA and RT-PCR

Total RNA of cells was isolated using Trizol reagent (Proteinbio, China). A TRUEscript RT kit + gDNA Eraser (Proteinbio, China) was to reverse-transcribe RNA to acquire cDNA. Genes targeted by quantitative PCR (2 × SYBR Green qPCR Mix with 100 × ROX, Proteinbio) were tested in triplicate. The primers for target genes were made from Realgene Company (Nanjing, China). Delta-delta cycle threshold (Ct) was used to detect target gene expressions, which were then standardized with GAPDH. The Forward and Reverse primers in PCR assays were: 5’-GCAGAGCTCGATTGTAGGGT-3’, 5’-CTGCAGTAGCTCCCATGTCC-3’ (ALG1L2); 5’-TCCTCCTCTTCAGTCTGCTAGT-3’, 5’-CCGGGTGGGTGACCATAGA-3’ (B3GNT3); 5’-GTCCCATTCAACCAGACGCTC-3’, 5’-GGGCACATAGAAGGGTCCTC-3’ (B3GNT8); 5’-GGTGGGCTGCTATAACTTGAC-3’, 5’-TCAGGTTGTTCTTTGCACTCTG-3’ (HS6ST3); 5’-TGCAACAGATCCTGTATGGCA-3’, 5’-CACCTGTCGAACAGCTCTGA-3’ (ST8SIA5); 5’-GGAGCGAGATCCCTCCAAAAT-3’, 5’-GGCTGTTGTCATACTTCTCATGG-3’ (GAPDH).

### RNA interference

The expression of B3GNT8 was silenced in two PC cell lines (CFPAC and MIA PaCa-2) using siRNA (GenePharma, China) at a concentration of 50 nM and applying Lipofectamine 3000 (Thermo Fisher Scientific) as transfection agent. B3GNT8 expression levels of PC cells were validated after cells transfection 48 h.

### Overexpression lentiviral transduction

The two types of lentiviruses carrying overexpressed human and mouse B3GNT8 gene were purchased from Corues Biotechnology Co., Ltd (Nanjing, China). A day before infection, PC cells were evenly seeded at 1 × 10^5^ cells per well in a 6-well cell culture plate. Subsequently, when the cell density reached 30%, added antibiotic-free complete culture medium containing diluted virus (MOI of 100) and the corresponding transfection enhancer (polybrene). After 18–20 h of infection, the culture medium was replaced with fresh DMEM complete medium. When the cell density reached 80%, puromycin was added to select cells overexpressing the B3GNT8 gene, and the surviving cells were cultured with half the puromycin concentration. At 48 h, the expression of B3GNT8 was verified at both the RNA and protein levels. Detailed experimental procedures can be found in our previous publication [12].

### Western blot

The total protein of PC cells was extracted with RIPA buffer supplemented with PMSF, and then boiled by adding 5 × SDS loading buffer (Proteinbio, China) after thorough mixing. Next, the proteins were separated by SDS-PAGE and then transferred to methanol-activated PVDF membranes (Bio-Rad). The membranes were blocked with QuickBlock buffer (Beyotime, China) for 30 min at room temperature and incubated with the relevant primary antibody (GAPDH: 1:2000, Proteinbio, China; B3GNT8: 1:1000, ThermoFisher,USA) at 4℃ for an overnight period. Afterwards, the membranes were washed 3 times with TBST for 10 min each, and then incubated with the relevant HRP-labeled secondary antibody (1:5000, Proteinbio, China) for 2 h at room temperature. Finally, the membranes were exposed and displayed using chemiluminescent HRP substrate (Biosharp, China) by the Invitrogen iBright1500 system. Membranes were trimmed based on marker positions and we retained the markers on each image.

### Functional experiments

#### Clone formation assay

PC Cells were cultured with complete medium and inoculated in 6-well plates (1000 cells/well) for 14 days of growth. Next, cells were then stained with 0.1% crystal violet for 20 min and washed with PBS after staining was completed. Only clones with a diameter of more than 1 mm were counted.

#### Transwell invasion assay

The upper chamber was coated with diluted Matrigel (BD Biosciences). Cell suspensions were prepared with serum-free DMEM and the cell concentration was adjusted to 2 × 10^5^/ml. Add 100 µL of cell suspension to the upper chamber and 500 µL of medium containing 10% FBS to the lower chamber and incubate the chambers at 37 °C in a 5% CO2 incubator for 24 h. Cells in the upper ventricles were gently wiped with cotton swabs, fixed with 4% paraformaldehyde for 10 min, then stained with 0.1% crystal violet for 5–10 min and washed three times with PBS. Ten fields of view were randomly evaluated under inverted microscopy (× 200). The experiments were done in triplicate.

#### Wound healing assay

Cells were planted in 6-well plates (7 × 10^5^ cells/well). After the cells have reached 90% fusion, straight scratches are made with a 200 µl pipette tip. The dropped cells were rinsed off with PBS and photographed and recorded at 0 and 48 h at the same locations. The relative wound area was calculated on Image J.

### Establishment of mouse pancreatic in situ tumor model

All animal experiments were reviewed and approved by the Institutional Animal Care and Use Committee (IACUC) of Nanjing Medical University (IACUC-2302020). Collect Pan02 cells grown to 90% confluency and count them, ensuring a cell concentration of 1 × 10^7^/ml. Dilute the high-concentration basement membrane gel with PBS at a 1:1 ratio. Administer inhalation anesthesia using isoflurane (RWD, China) on mice. Then inject these cells at a dose of 50 µL into the head of wild-type C57BL/6 mice pancreas. After tumor cell inoculation, continue to house the mice in a sterile animal room. 4 weeks later, the mice were euthanized for the purpose of assessing the growth of pancreatic tumors and the presence of liver metastasis. Subsequently, the collected samples underwent Hematoxylin and Eosin (HE) staining. For specific procedural details, please refer to our previous publication [13]. The animals are breed in accordance with the protocols approved by the Animal Protection and Use Committee of Nanjing Medical University, and they all receive humane protection with the standards in the “*Guidelines for Laboratory Animal Care and Use*”. This study was carried out in compliance with the ARRIVE guidelines [14].

### Multi-label tissue staining and image analysis

Multi-label immunohistochemical staining technique based on Tyramide Signal Amplification (TSA, Tyramide Signal Amplification) was used to stain the FFPE (formalin-fixed paraffin-embedded) samples. We conducted multi-label composite staining on the same sample using primary antibodies of the same genus origin, but with different specificities. Using a digital pathology image panoramic scanning system to obtain pathology image information for the entire slice by scanning the entire spot [15]. We defined the tumor cell aggregation as a “tumor nest” and used the nest as the basic unit for subsequent calculations. Quantitative analysis of the scans was performed using the HALO Highplex FL (Indica Labs; Albuquerque, NM) analysis module of the pathological image analysis software Halo.

### Statistical analysis

All data were analyzed using SPSS 25.0 statistical software and GraphPad Prism 7. The t-test and two-way ANOVA were used to compare between groups. Screening for differential genes via the Wilcoxon test. Association between B3GNT8 expression and clinical pathological factors was explored via χ2 test. In PC patients, correlation between the survival rates and B3GNT8 expression was examined via Kaplan–Meier analysis and log-rank test. Univariate survival analysis was finished with a Cox proportional hazard regression model. All statistical tests were two-tailed at the significant level *P* < 0.05, shown as *P < 0.05, **P < 0.01, ***P < 0.001.

## Results

### Identification of differential glycosylation related genes in PC

**Fig. 1:**
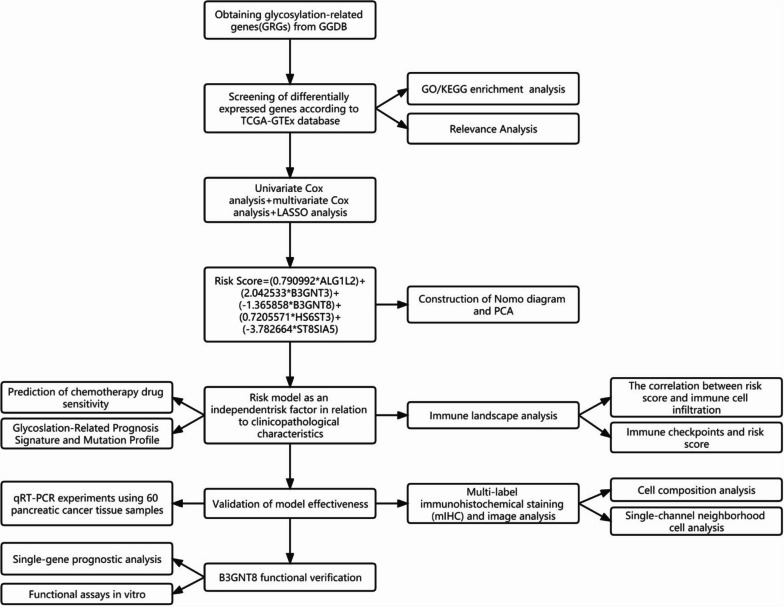
The workflow of this study

**Fig. 2:**
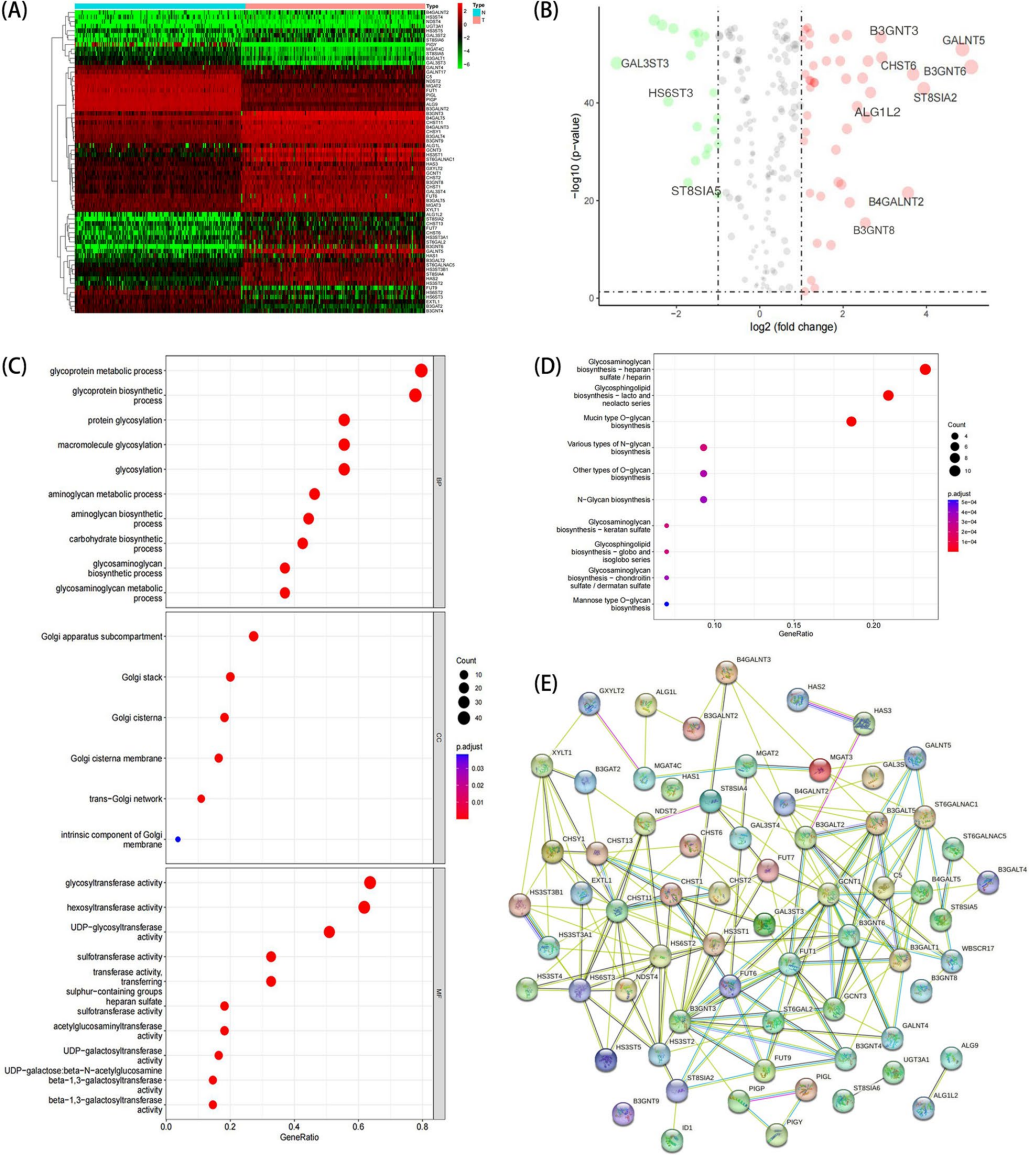
Identification of differential glycosylation related genes (GRGs). **A** Heatmap of 66 DE-GRGs in TCGA and GTEx joint database. **B** Volcano map of glycosylation-related genes in TCGA and GTEx joint database. The criteria were *P* < 0.05 and |logFC|> 1. **C** GO analysis of DE-GRGs, including BP (biological process), CC (cellular component) and MF (molecular function). **D** KEGG pathway analysis. **E** Constructing protein interaction networks for DE-GRGs using STRING website

To investigate whether the abnormal glycosylation related gene have a relationship with PC risk and tumor immune environment, the process we designed is depicted in Fig. 1. We used a dataset combining expression data and clinical information of PC patients downloaded from the TCGA database and GTEx database. 199 Glycogenes were selected from the online glycogene database (GGDB, https://acgg.asia/ ggdb2/). Glycogenes include genes associated with glycan synthesis such as glycosyltransferase, sugar nucleotide synthases, sugar-nucleotide transporters, sulfotransferases, etc. The GRG expressions were extracted from the dataset and subsequently subjected to differential analysis. Among 179 PC samples and 169 pancreatic normal samples, 66 differentially expressed GRGs contained 20 down-regulated genes and 46 up-regulated genes were identified using the Wilcoxon test (Fig. 2A, B). To further detect the biological significance of glycosylation in the pathogenesis and progression of PC, we conducted GO and KEGG pathway analyses. GO enrichment analysis reveals that differential genes were found to significantly participate in the formation of cellular components, specifically including subcompartments, stacks, and cisternae within the Golgi apparatus. The molecular functions included glycosyltransferase activity, hexosyltransferase activity and UDP − glycosyltransferase activity. The biological processes included glycoprotein biosynthetic and metabolic processes and protein glycosylation (Fig. 2C). KEGG pathway analysis indicated that the majority of pathways associated with differentially expressed genes were significantly enriched in glycosaminoglycan biosynthesis, mucin type O-glycan biosynthesis, and various types of N-glycan biosynthesis (Fig. 2D) [16, 17]. Moreover, we utilized STRING tool to forecast protein interactions among the differentially expressed genes, these genes involved in glycosylation display a close association with each other, as demonstrated in Fig. 2E.

### Construction and validation of the glycosylation-based prognostic risk model

**Fig. 3:**
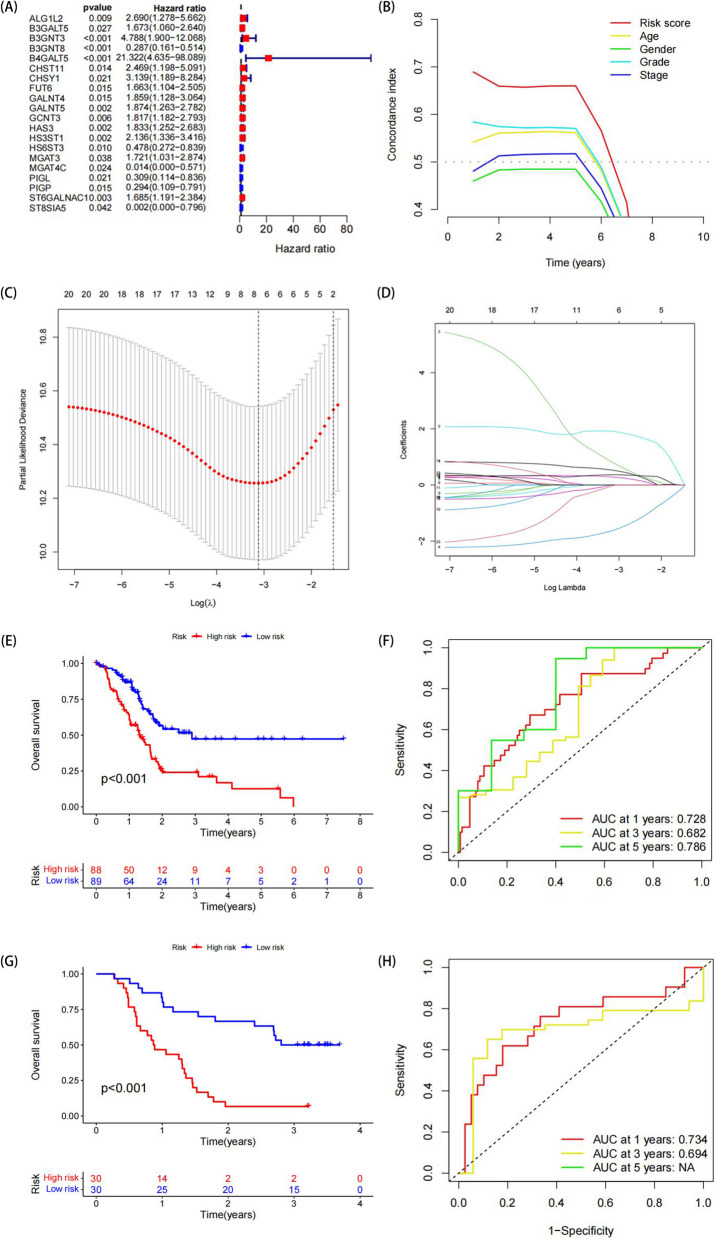
Screening GRGs to construct a prognostic model and verifying its feasibility. **A** Univariate Cox regression analysis to select prognosis related GRGs. **B** Predictive accuracy of C-index detection prognostic model. **C** Lasso regression analysis to select the best parameters. **D** The association with regression coefficients and log-transformed lambda. **E** Kaplan–Meier curves were plotted in the entire TCGA cohort after distinguishing between high- and low-risk groups according to the model. **F** The ROC analysis at 1,3,5 years of overall survival in the entire TCGA cohort. **G** Kaplan–Meier curves were plotted in the tissues from the first affiliated Hospital of Nanjing Medical University after distinguishing between high- and low-risk groups according to the model. **H** The ROC analysis at 1,3,5 years of overall survival in the PC patient tissues

To develop the prognosis-related GRGs risk model in PC, survival data and expression profiling of PC patients in the TCGA cohort were screened by univariate Cox regression analysis. A total of 20 genes were identified as prognosis-related GRGs, the majority of which have been previously linked to tumor development. (Fig. 3A). The C-index was used to assess the predictive accuracy of the prognostic model, which performed better than other clinical parameters (Fig. 3B). LASSO regression analysis and multivariate Cox analysis were employed to identify five GRGs-associated genes that were then used to build the prognostic risk models (Fig. 3C, D). The formula for calculating the risk score was finalized as follows: risk score = (0.79 × ALG1L2) + (2.04 × B3GNT3) + (-1.36 × B3GNT8) + (0.72 × HS6ST3) + (-3.78 × ST8SIA5). After calculating each patient's risk score in the TCGA-GTEx set, they were categorized into high- and low-risk groups based on the median risk score. Kaplan–Meier analysis revealed that patients in the high-risk group had a worse prognosis than those in the low-risk group (Fig. 3E). Using the AUC value, we evaluated the prognostic ability of the risk model comprising the five genetic signatures. The AUC values for predicted risk scores for OS at one, three, and five years were 0.728, 0.682, and 0.786, respectively, in the TCGA-GTEx dataset (Fig. 3F). Subsequently, a series of validations of the risk model's validity was conducted on a total of 60 pancreatic cancer tissues obtained from the Pancreatic biobank in the First Affiliated Hospital of Nanjing Medical University. Kaplan–Meier analysis revealed a significantly poor prognosis in high-risk patients (Fig. 3G), and ROC curve analyses showed that the AUC value for the one- and three-year OS was 0.734 and 0.694, respectively (Fig. 3H). Since all the samples were collected after 2019, we lacked five-year survival information. In both TCGA and validation data, the model gene's expression significantly differed in the high- and low-risk groups. Subsequently, we supplemented data from the GEO database as an external validation set (GSE28735, GSE57492, GSE62452) to further verify the feasibility of the model. The results have been added in Supplementary Fig. 5, indicating that within the GEO database, the prognostic model established in this study can also effectively assess patients' prognostic outcomes. These results suggest that the prediction model has specificity and accurate predictive ability for pancreatic cancer patients.

### Risk model as an independent risk factor with respect to clinicopathological features and survival prognosis

**Fig. 4:**
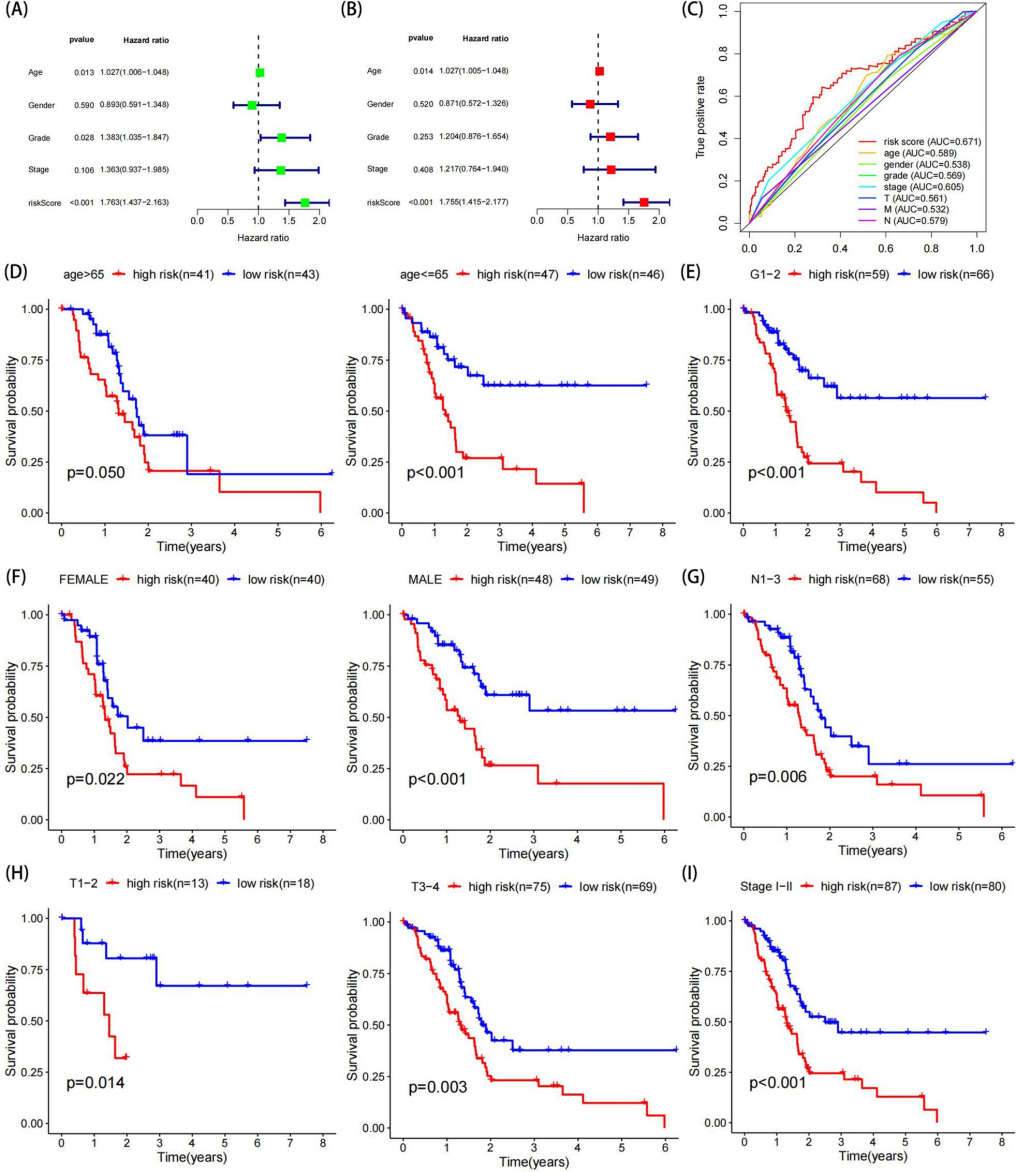
Stratification of patients according to clinicopathological parameters for further validation of survival analysis. **A** Univariate Cox regression analysis demonstrated that risk score was associated with pancreatic cancer prognosis. **B** The prognostic model was an independent factor for pancreatic cancer. **C** ROC analysis of the prognostic model and other clinicopathologic parameters. **D**, **I** KM survival analysis of risk score and clinical parameters

The Wilcoxon Rank Sum test revealed that ALG1L2, B3GNT3, and B3GNT8 were expressed at higher levels in tumor tissues, while HS6ST3 and ST8SIA5 were expressed at lower levels in tumor tissues (all *P* < 0.01; Supplementary Fig. 1). We conducted univariate and multivariate analyses to assess the values of clinical parameters, pathological parameters, and risk scores on prognosis. The results showed that age, tumor grade, and risk score were related to OS (Fig. 4A), and risk score was identified as an independent risk factor for PC (HR 1.755, 95% CI 1.415–2.035, *p* < 0.001) (Fig. 4B). ROC analysis was used to evaluate the sensitivity and specificity of the model, and the AUC was 0.671, which was larger than that of other pathological and clinical factors, including age, gender, grade stage, and TNM stage (Fig. 4C). Furthermore, patients were subdivided into clinicopathological subgroups for further survival analysis. The results demonstrated that the risk model was significant for OS in the vast majority of clinical subgroups, except for grade 3–4, stage III/IV, M0/1, and N0 (Fig. 4D-I).

### Establishment of overall survival nomograms and principal component analysis (PCA)

**Fig. 5:**
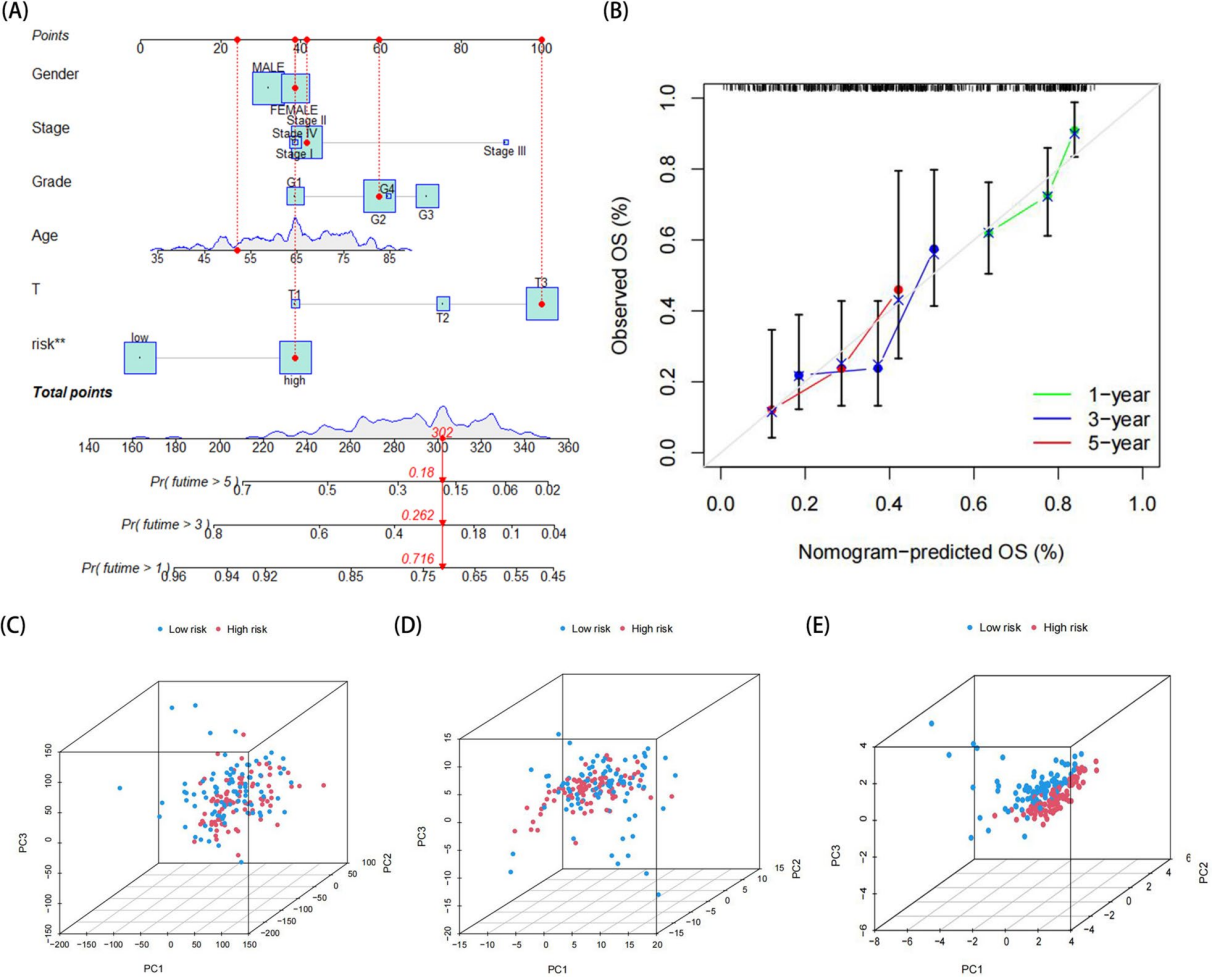
Construction of Nomo diagram and PCA analysis. **A** The 1-year, 3-year and 5-year survival predicted by the nomogram based on the prognostic model. **B** Calibration plots to evaluate the consistency between the predicted and actual 1-year, 3-year and 5-year survival. **C** PCA analysis of each genome in the TCGA cohort. **D** PCA analysis based on all genes related to glycosylation. **E** PCA analysis of risk model genome

To better elucidate the survival risks of PC patients, we developed prognostic nomogram that combined prognostic models and clinicopathological features to investigate the roles of the five risk genes in 1-, 3-, and 5-year OS (Fig. 5A). The calibration curves of the 1- to 5-year OS rate displayed a promising level of agreement between observations and predictions (Fig. 5B). In prognostic analysis, multiple variables are often intercorrelated. Principal Component Analysis (PCA) can be used to reduce data complexity and maximize the information retained by projecting the data onto lower-dimensional principal components based on the study of correlations between individual variables. PCA is commonly used to show the risk distribution of a certain population based on risk and differential gene sets, as well as genome-wide expression sets [12]. PCA of the entire gene expression profile revealed that the isolation of risk states was unclear (Fig. 5C). With regard to all glycosylation-associated genes, it was not possible to separate low- and high-risk populations into two distinct groups (Fig. 5D). However, with the risk model genome, the two risk groups were significantly different, indicating a clear separation (Fig. 5E).

### Immunological differences between high- and low- risk groups

**Fig. 6:**
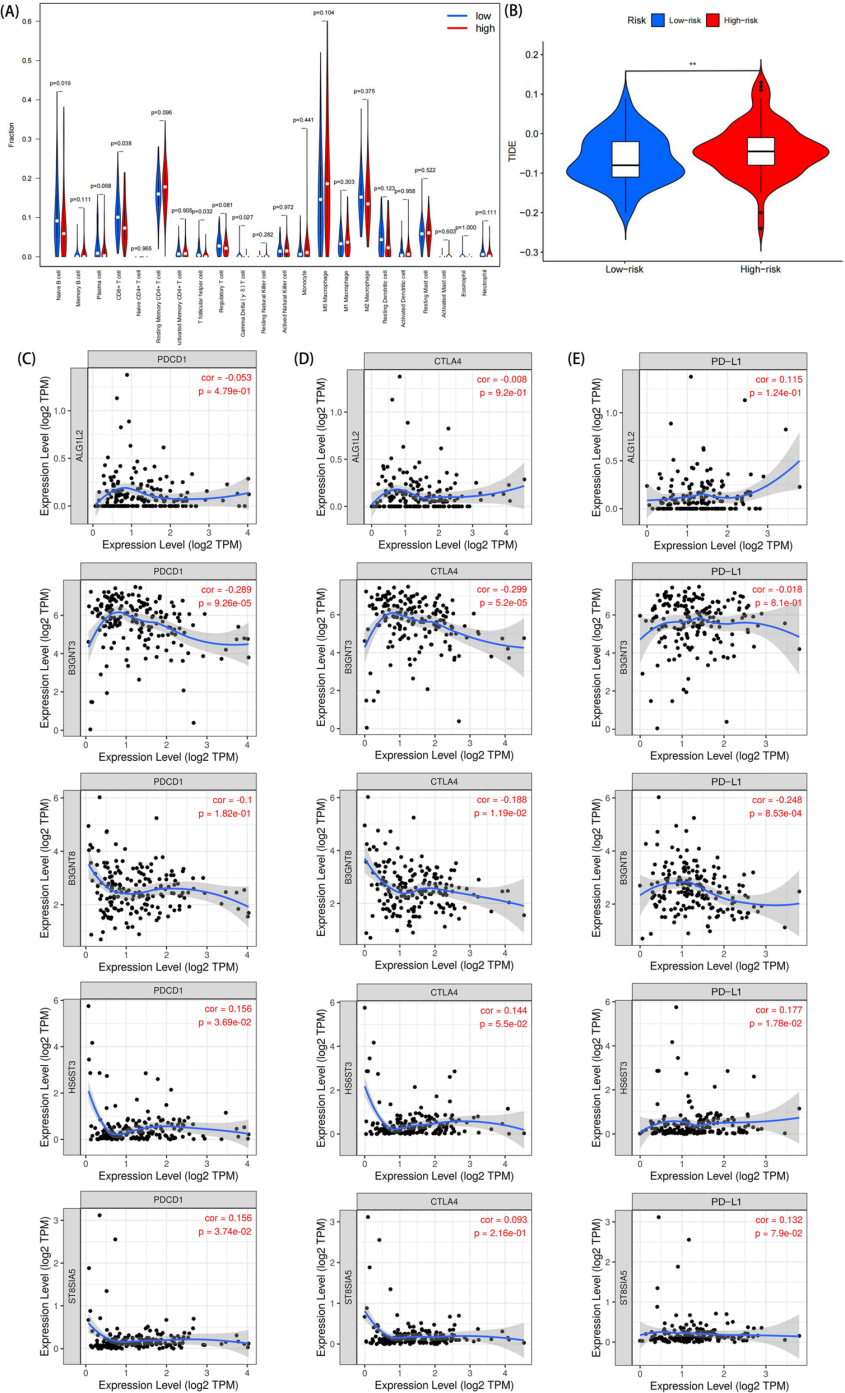
Risk score is associated with immune checkpoints. **A** Violin plot of the difference in immune cell infiltration between high- and low- risk groups. **B** TIDE scores for high and low risk groups. (C)-(E) Association between risk score and common immune checkpoints including PDCD1 **C** CTLA4 **D** and PD-L1 **E**

We evaluated the extent of tumor lymphocyte distribution and calculated the scores of different lymphocyte infiltration fractions in both high- and low-risk groups. Meanwhile, we compared the differences in 22 immune-related cells in two risk groups (Fig. 6A). Four immune cells showed differences between the risk groups, including gamma delta T cell, CD8^+^ T cell, naïve B cell and T follicular helper cell. Supplementary Fig. 2 shows specifically the differences in expressions of four tumor infiltrating lymphocytes between the two risk groups. The TIDE (Tumor Immune Dysfunction and Exclusion) score is a scoring system that measures the immunosuppressive and immunoreactive signals in the tumor microenvironment, it could predict the response of tumors to immunotherapy. The low TIDE score observed in the low-risk group indicates that immunotherapy may be a viable treatment option for this subgroup and could represent a novel therapeutic avenue (Fig. 6B). Immunotherapy has emerged as a promising approach in the treatment of PC. We assessed the association between risk score and common immune checkpoints (ICPs), including PDCD1, CTLA4 and PD-L1. Five genes incorporated in the prognostic model are closely associated with these ICPs (Fig. 6C, D, E). Among the five genes, B3GNT3 and B3GNT8 showed higher correlation with ICPs, suggesting the B3GNT family may play a critical role in the tumor immune process.

### Panoramic analysis by multicolor fluorescence immunohistochemistry

**Fig. 7:**
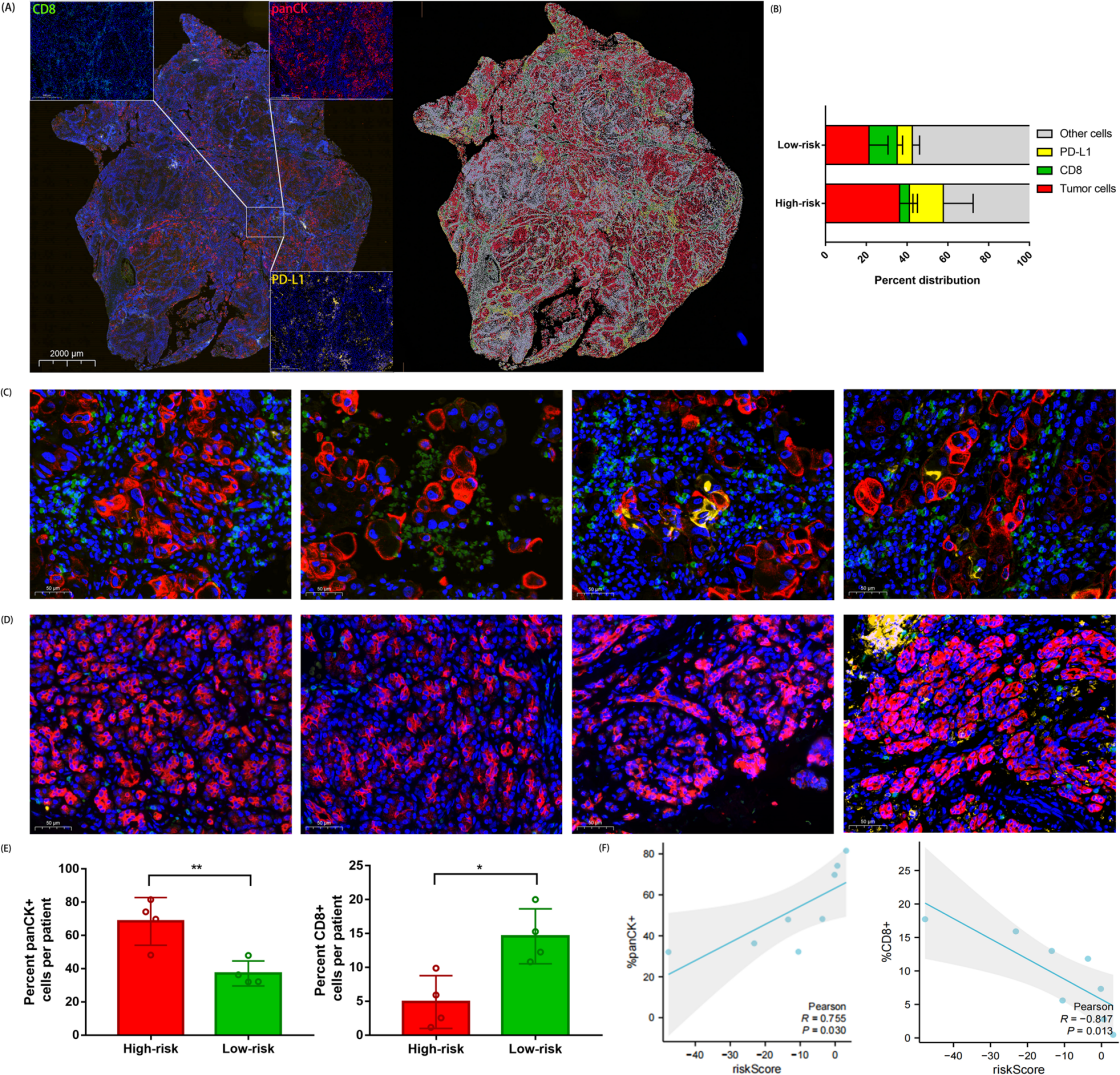
Less tumor cells and more tumor infiltration CD8 + T cells were observed in GRGs low risk group. Representative staining of cells in PC, each of the individual markers in the composite image after spectral unmixing, together with the DAPI nuclear marker (pseudocoloured blue) and the autofluorescence signal (pseudocoloured black). **A** Relative distribution of all analysed cell phenotypes in PC and uninvolved pancreatic tissue samples and pairwise comparisons of the percentage of CD8 + and PD-L1 + cells. **B** Comparison of the difference in the percentage of single channel positive cells(panCK + , PD-L1 + , CD8 +) in high and low risk groups. **C** Representative mIHC images of 4 patient samples in the low-risk group. **D** Representative mIHC images of 4 patient samples in the high-risk group. **E** Comparison of the difference in the percentage of single channel positive cells (panCK + , PD-L1 + , CD8 +) in high and low risk groups. **F** Correlation between risk score and percentage of single channel positive cells

We selected paraffin tissues from four patients in both high- and low-risk groups for multiplex immunohistochemistry (mIHC) staining using multi-color fluorescence immunohistochemistry. The corresponding information of each cell type and its staining channel is shown in Table S1. Four different fluorescence clusters were simultaneously captured into a composite image, which was then separated into images representing each fluorescence cluster and the nuclear stain DAPI. The panoramic scanning results were quantitatively analyzed using the pathology image analysis software Halo, and the analysis results summary is shown (Fig. 7A). We analyzed the panoramic distribution of cell composition, and the results indicated significant differences in cell distribution between the high-risk and low-risk groups. The proportion of tumor cells in the high-risk group was significantly higher than that in the low-risk group. In contrast, the proportion of CD8-positive cells was higher in the low-risk group, indicating a higher level of cytotoxic T cell infiltration and suggesting a better prognosis in the low-risk group (Fig. 7B). Furthermore, we conducted a correlation analysis between the proportion of single-channel positive cells and the risk score. Fig. 7C and D showed representative images randomly selected from different risk groups. Four patients in the low-risk group showed more CD8 + T cell infiltrations around the tumor tissue (Fig. 7C). In contrast, there were fewer CD8 + infiltrations in the four patients from the high-risk group (Fig. 7D). The results of single-channel analysis demonstrated that the proportion of panCK-positive cells was higher in the high-risk group, and the degree of CD8 + cell infiltration was lower, indicating immune exhaustion and poorer prognosis in patients (Fig. 7E). Consistent with previous trend, the risk score showed a positive correlation with the proportion of tumor cells and a negative correlation with T cell infiltration (Fig. 7F). The above results indicated that consistent with the predicted trends in the TCGA database, with the increase of the risk score, the immune infiltration in the tumor tissue decreases, and the prognosis becomes worse.

### Single-channel neighborhood cell analysis

**Fig. 8:**
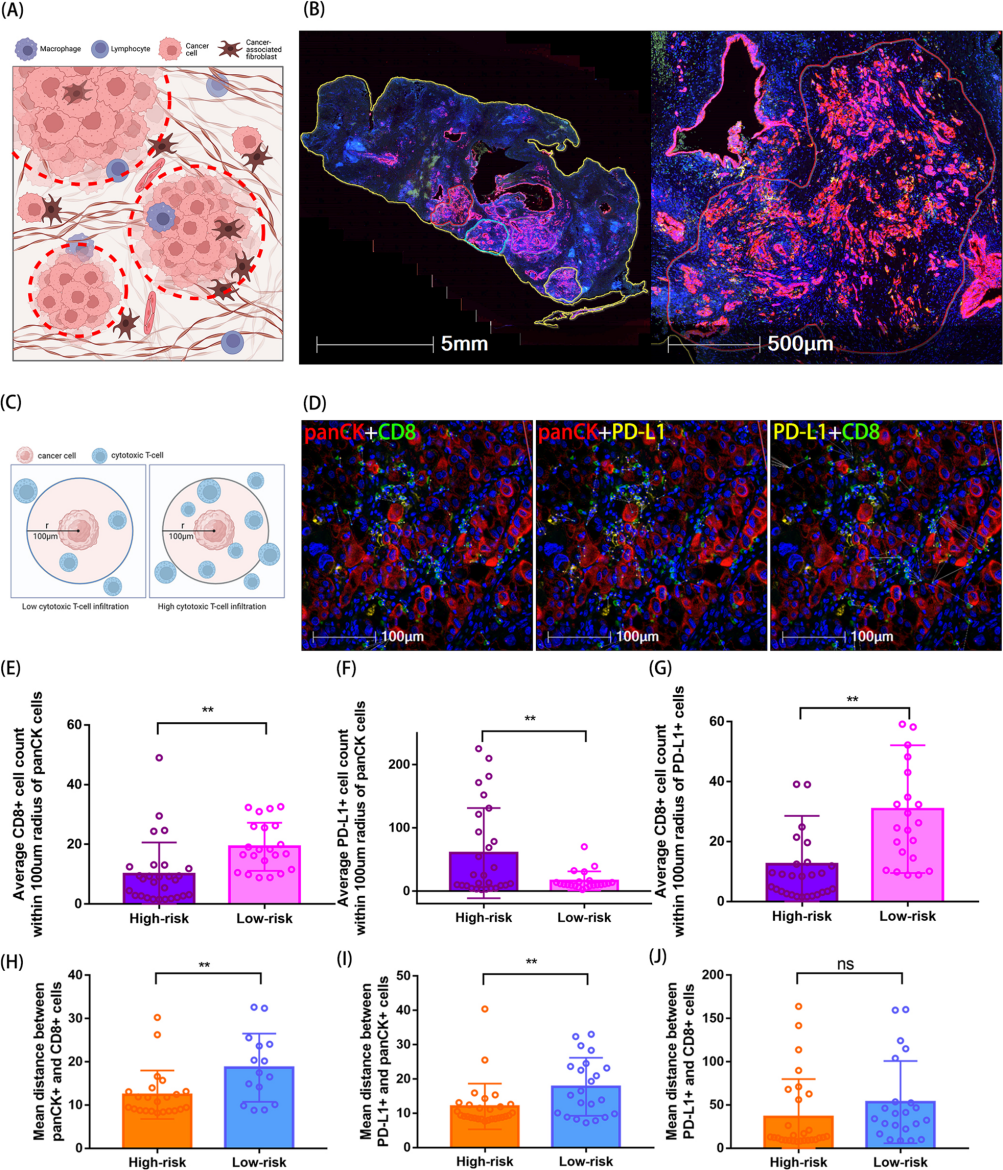
Single-channel neighborhood cell analysis. **A** Schematic diagram of “cancer nest”. **B** Representative schematic of “cancer nest”. **C **Schematic diagram of cell number analysis within the 100 μm range. **D **Representative schematic of nearest distance analysis. **E** Comparison of the number of CD8 + cells in the 100um range of panCK-positive cells. **F **Comparison of the number of PD-L1 + cells in the 100um range of panCK-positive cells. **G **Comparison of the number of CD8 + cells in the 100um range of PD-L1-positive cells. **H** Analysis of the average closest distance between panCK + cells and CD8 + cells. **I** Analysis of the average closest distance between panCK + cells and PD-L1 + cells. **J **Analysis of the average closest distance between PD-L1 + cells and CD8 + cells

To further analyze the crosstalk between tumor cells and CD8 + T cells in PC, we defined the sites where tumor cells cluster as "tumor nest" and used this as the basic units for annotation and subsequent calculations. The schematic of tumor nest is shown in Fig. 8A, and representative images of tumor nest in the samples are shown in Fig. 8B. In order to influence the function of tumor cells and to transmit excitatory or inhibitory signals, immune cells must be in close proximity or in contact with tumor cells [18]. To characterize the spatial distribution of cells within PC, we selected circular regions with a radius of 100 μm around each tumor cell and recorded the average number of other single-positive channel cells present in each region (Fig. 8C). Representative images are shown in Fig. 8D. We observed that in high-risk group patients, the number of CD8 + T cells around tumor cells was lower than that in the low-risk group (Fig. 8E), but the number of PD-L1-positive cells was higher (Fig. 8F). At the same time, there was a significant difference in the number of PD-L1-positive cells around CD8 + T cells. In the high-risk group, there were fewer CD8 + T cells around PD-L1-positive cells (Fig. 8G). We also calculated the shortest distance between single-positive cells in pairwise manner, which can better reflect the degree of cell infiltration. In the high-risk group, the number of CD8 + T cells infiltrating around tumor cells decreased, and the average distance between tumor cells and T cells was also shortened, indicating that CD8 + T cells near the tumor cells may be exhausted (Fig. 8H). Interestingly, although the number of PD-L1-positive cells around tumor cells within a radius of 100 μm was higher in the high-risk group than in the low-risk group, the average distance between the two types of cells was closer in the high-risk group (Fig. 8I). There was no significant difference in the distance between cytotoxic T cells and PD-L1-positive cells between the high-risk and low-risk groups (Fig. 8J). Overall, cell number analysis within a certain range and minimum distance could evaluate the infiltration of cells in tumors from different perspectives. The results showed immune infiltration microenvironment is worse in the high-risk group.

### Potential chemotherapy drugs screening

R package “pRophetic” was used to predict the IC50 of 35 commonly used chemotherapeutic agents within two risk groups [13]. The principle of the algorithm is to apply gene expression data from a large set of cancer cell lines, combine them with drug sensitivity data to build a statistical model, and then apply the model to clinical tumor biopsy samples. There is a correlation between our risk model and the sensitivity to 12 chemotherapeutic agents. Among them, the risk model was predicted to positively correlate with six agents (Axitinib, CEP-701, EHT 1864, MP470, Nilotinib, Phenformin) (Supplementary Fig. 3A). It is also corresponded to higher IC50 within the high-risk group, indicating that patients in the low-risk group may have a better outcome with these six drugs (Supplementary Fig. 3B). The risk model is negatively related to the remaining six agents (Epothilone B, JW-7–52-1, GW843682X, Paclitaxel, BI-2536, EKB-569) and corresponds to lower IC50 in the high-risk group, suggesting that the high-risk patients have more sensitivity to these six agents.

### Glycosylation-related risk model and mutation profile

Tumor mutation burden (TMB) is defined as the total number of mutations in each coding region of the tumor genome. Current studies have shown that TMB has a good predictive value for immunotherapy of a wide range of tumors. We grouped 178 PC patients according to their TMB scores, the mutation levels of patients in the sample were then analyzed in combination with high and low risk levels. The results indicated the best prognosis was observed in the low-risk and low-mutation groups. As the risk score increased and the tumor mutation load increased, the prognosis of patients became significantly worse and survival was obviously shorter (Supplementary Fig. 4A). Somatic mutation data were utilized to evaluate the TMB of patients. The mutation frequencies in both groups were almost the same, and the top ten were KRAS > TP53 > SMAD4 > CDKN2A > TTN > MUC16 > RNF43 > HECW2 > TNXB > RYR1, but the mutation frequency was significantly higher in the high-risk group (Supplementary Fig. 4B, C).

### B3GNT8 serves as a protective factor in risk score and PC patient survival

**Fig. 9:**
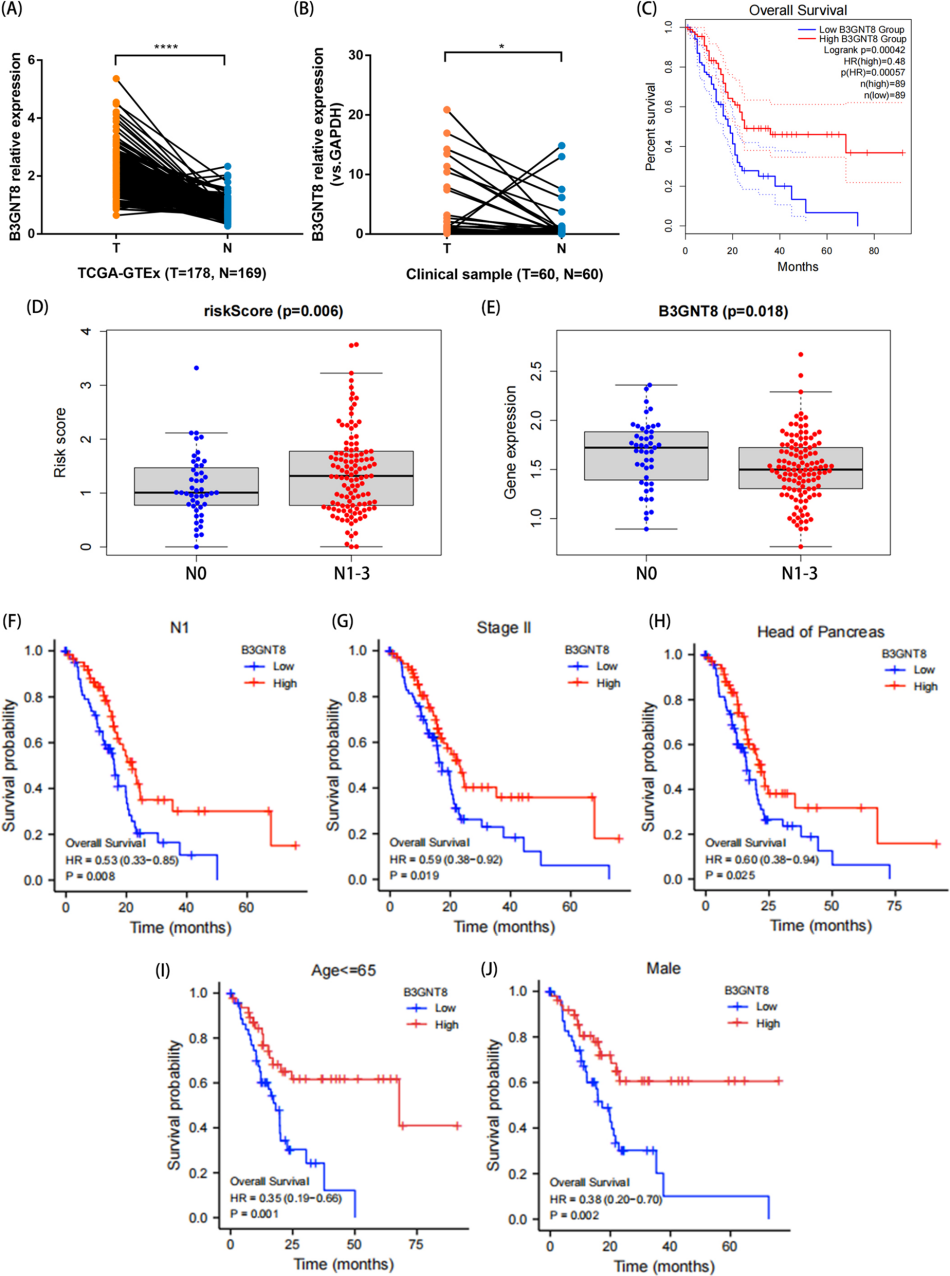
Correlation analysis of B3GNT8 expression with subgroups of clinical parameters. **A** B3GNT8 expression in PC patients samples from TCGA-GTEx database. **B** B3GNT8 expression in PC patients samples from the first affiliated Hospital of Nanjing Medical University. **C** Kaplan–Meier plots showed the correlation between B3GNT8 expression and overall survival in TCGA database. **D** Relationship between risk score and different N stages. **E** Relationship between B3GNT8 and different N stages. **F**, **J** Survival analysis of B3GNT8 expression in lymph node metastasis(N1), Stage II, pancreatic head carcinoma, age <  = 65 and male subgroups

In previous studies, the prognostic value of a signature consisting of five glycosylation-related genes has been demonstrated. Among the five model genes, B3GNT8 was highly expressed in PC tissues both in the public database TCGA-GTEx and the verification results from single center (Fig. 9A, B). However, during the risk score calculation process, our multiCox analysis revealed a negative coefficient and HR < 1 for B3GNT8, indicating that it may serve as a protective factor against PC. Kaplan–Meier plots also showed the correlation between B3GNT8 expression and overall survival in TCGA database. Consistent with the trend predicted by the risk model, overall survival was longer in patients with high expression of B3GNT8 (Fig. 9C). Next, we analyzed the correlation between risk score and B3GNT8 expression with clinical parameters. Risk scores were higher in patients with stage N1-3, indicating a positive correlation between risk scores and lymph node infiltration (Fig. 9D). In contrast, B3GNT8 was expressed at a lower level in patients with lymph node invasion (Fig. 9E). We then performed a subgroup clinical parameter survival analysis for this gene. In clinical subgroups, patients with high B3GNT8 expression had a better prognosis in lymph node metastasis(N1), stage II, pancreatic head carcinoma, age <  = 65 and male subgroups (Fig. 9F-J). Hence, B3GNT8, which is highly expressed in tumors but acts as a cancer inhibitor, is well worth further investigation.

### B3GNT8 plays a role in inhibiting pancreatic cancer growth and metastasis

**Fig. 10:**
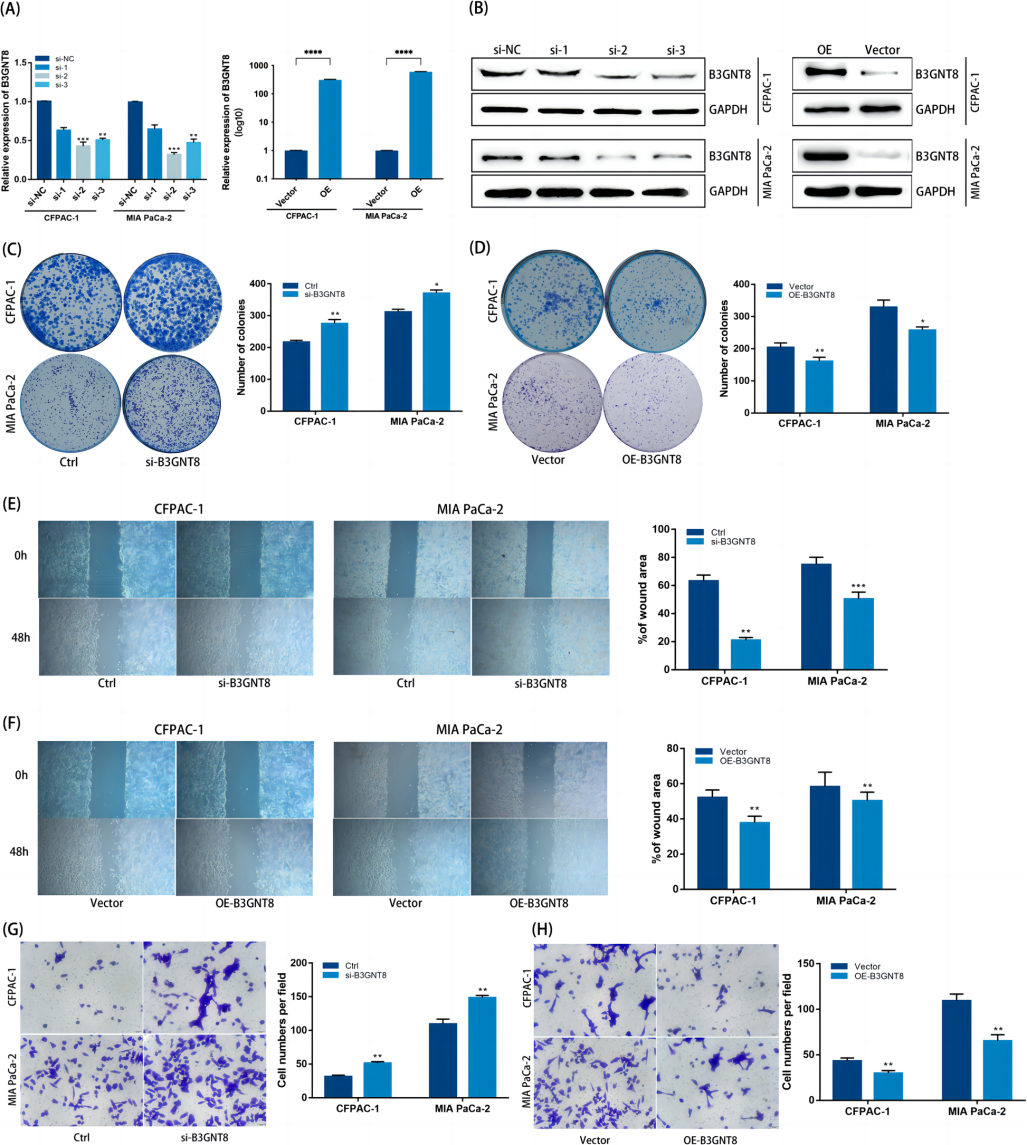
B3GNT8 functional verification. **A** Expression of B3GNT8 mRNA in CFPAC-1 and MIA PaCa-2 cells after knocking down and overexpressing B3GNT8. **B** Western blot showed B3GNT8 protein expression in CFPAC-1 and MIA PaCa-2 cells after knocking down and overexpressing B3GNT8. The samples are from the same experiment, and the gels/blots were processed in parallel. **C** Effects of si-B3GNT8 on clonogenic ability of CFPAC-1 (up) and MIA PaCa-2 (down) cells. **D** Effects of OE-B3GNT8 on clonogenic ability of CFPAC-1 (up) and MIA PaCa-2 (down) cells. **E** Wound-healing assay showed the migration of CFPAC-1 (left) and MIA PaCa-2 (right) cells were promoted by B3GNT8 knockdown. **F** Wound-healing assay showed the migration of CFPAC-1 (left) and MIA PaCa-2 (right) cells were inhibited by B3GNT8 overexpression. **G** Transwell assay showed the invasion of CFPAC-1 (up) and MIA PaCa-2 (down) cells were promoted by B3GNT8 knockdown. **H** Transwell assay showed the invasion of CFPAC-1 (up) and MIA PaCa-2 (down) cells were inhibited by B3GNT8 overexpression

In order to investigate the exact function of B3GNT8 in the development of PC and to further validate the accuracy of the model, we investigate the B3GNT8 functions in vitro. Three different siRNAs were used to knock down B3GNT8, and siRNA-2 demonstrated more than 50% depletion of B3GNT8 in CFPAC-1 and MIA PaCa-2 cell lines. Lentivirus was used for overexpression of B3GNT8, and validation was performed at the RNA levels to confirm the levels of knockdown and overexpression (Fig. 10A). Western blot detected the corresponding B3GNT8 protein levels, which was consistent with mRNA levels (Fig. 10B). Knockdown of B3GNT8 significantly promoted the clonogenic ability of CFPAC-1 and MIA PaCa-2 cell lines, while overexpressing B3GNT8 inhibited the proliferation of PC cells (Fig. 10C, D). The transwell and wound healing assays demonstrated that knockdown of B3GNT8 enhanced the invasive and migratory potential of PC cells (Fig. 10E, G). On the contrary, overexpression of B3GNT8 inhibited the invasive and migratory abilities of PC cells. (Fig. 10F, H).

Next, we randomly divided the mice into two groups, with 3 mice in each group. One group was injected with Pan02 cells stably overexpressing B3GNT8 lentivirus, and the other group was injected with Panc02 cells stably transduced with negative control lentivirus. Samples were collected at the fourth week after injection to observe the development of in situ tumors and metastasis in the mice. As shown in Supplementary Fig. 6A, compared to the negative control, the group with elevated B3GNT8 expression displayed visibly reduced sizes of pancreatic in situ tumors, and there was also a significant decrease in the number of liver metastatic lesions (Supplementary Fig. 6B). Subsequently, we performed HE staining on the collected tissue samples to confirm the occurrence of in situ tumor formation and liver metastasis, as depicted in Supplementary Fig. 6C and D. The corresponding statistical analysis is presented in Supplementary Fig. 6E and F. The preliminary results above demonstrate that, consistent with the in vitro experimental findings, B3GNT8 can exert an inhibitory effect on tumor growth and metastasis in vivo.

## Discussion

Pancreatic cancer is a highly malignant tumor with a poor prognosis, exhibiting nearly equivalent morbidity and mortality rates [19]. The poor prognosis of PC is attributed to the absence of biomarkers for early detection, resulting in 90% of PC patients being diagnosed at a late stage at diagnosis [20, 21]. In addition, PC is not sensitive to chemotherapy and immunotherapy, and even combination therapy fails to ameliorate unfavorable clinical outcomes [22]. Therefore, identifying suitable biomarkers for PC diagnosis and developing novel tools to guide precision therapy are crucial in PC treatment.

Glycosylation is the enzymatic process of linking sugars to proteins, lipids, and other glycans, and this major post-translational modification (PTM) occurs in the endoplasmic reticulum/Golgi lumen of all cells and is mediated by the coordinated action of different glycosyltransferases and glycosidases [23]. Glycosylation is the basis of various biological processes and small changes in glycan structure severely affect cell biology, causing pathophysiology and development [24], such as cancer [8]. Malignant transformation is highly associated with aberrant glycosylation [25]. Previous studies have found that glycosylation-related genes are associated with the prognosis of patients with breast [26], ovarian [27], liver [28] and cervical cancer [29]. However, current studies on glycation in PC mainly focus on the influence of individual glycation type or single glycation enzyme on tumor progression [30, 31], and there are no relevant reports on the expression status of numerous glycation related genes in PC and their relationship with PC microenvironment [32]. Gupta et al. summarized the expression of 207 glycation genes in TCGA database and identified 6 glycation genes (B3GNT3, B4GALNT3, FUT3, FUT6, GCNT3 and MGAT3) that played a unique role in the pathogenesis of PC [33]. Their functions were investigated using the CRISPR/ cas9 based KD system. The key role of O- and N- linked glycosylation in PC progression was finally demonstrated and the mechanism of GCNT3 action in PC was described. However, this study included only 149 tumor patients and 3 paracancer tissues for analysis, and mainly focused on the function of glycosylation genes on tumor cells. In addition, Yousra et al. performed a bioinformatic analysis including 169 glycosyltransferase RNA sequencing data from resected and unresectable 74 patient-derived xenografts (PDX) and constructed a glyco-signature consisting of 19 genes [34]. Their research also has limitations including insufficient sample size and too many model genes. Hence, we first acquired GRGs through the GGDB and tested their expressions from TCGA-GTEx joint database, which included 178 PC samples and 169 normal control samples. Based on the univariate and multivariate cox regression analysis, 5 genes associated with GRG were identified for prognostic modeling. In order to verify the reliability of the model, we randomly selected 60 pairs of pancreatic tumors and para-cancer samples from the first affiliated Hospital of Nanjing Medical University to validate the prognostic performance of the GRGs signature. Moreover, the five GRGs prognostic model were consisted of 3 elevated expressed (ALG1L2, B3GNT3, B3GNT8) and two decreased genes (HS6ST3, ST8SIA5). Notably, B3GNT8 was negatively correlated with risk score, however, the prognosis of PC patients was better. Hence, we chose B3GNT8 for functional experiments to further verify the accuracy and practicability of the risk score. B3GNT8 was initially cloned in 2004 [35]. Subsequently, in 2005, a team of Japanese researchers led by Akira Seko demonstrated that B3GNT8 could form heterodimers with B3GNT2 in vitro, resulting in an enhancement of catalytic activity [36]. Building on this, a study conducted in 2010 by Shen unveiled that B3GNT8 plays a role in regulating matrix metalloproteinase 2 (MMP-2) and tissue inhibitor of metalloproteinase 2 (TIMP-2) in gastric cancer cells [37]. These findings propose that B3GNT8 might offer potential as a therapeutic target for gastric cancer. Based on the risk scores, we discovered the risk model has relationship with tumor infiltrating lymph cells, immune checkpoint, chemotherapy drug sensitivity, immune escape and tumor mutational load in PC. However, further verification is still required to establish the correlation between the risk model and tumor immune microenvironment.

PC is one of the most immune-resistant tumor types, exhibiting an immunologically "cold" tumor microenvironment (TME) [38]. This indicates a lack or dysfunction of adaptive T cell immunity and resistance to checkpoint blockade [23]. To date, monotherapy with immune modulators has been proven ineffective in PC clinical settings, indicating a multimodal approach targeting the immune therapy resistance mechanism [39]. Personalized immunotherapy strategies based on PC genetic and phenotypic heterogeneity for individual patients may offer new insights [40]. Therefore, to identify potential immune features, we performed multi-marker immunohistochemistry staining on 4 samples selected randomly from both high- and low-risk groups. We labeled tumor cells with panCK, CD8 and PD-L1 to observe the expression differences of these markers in PC tissues. The results showed more tumor cell and less CD8 + T cells were consisted in high-risk samples. Moreover, we performed a neighboring cell analysis, which was divided into cell count within a 100 μm range and analysis of the nearest cell-to-cell distance. We observed an interesting phenomenon that, although the infiltration of cytotoxic T cells was reduced, their distance to tumor cells was closer. Mature CD8 + T cells, also known as cytotoxic T cells, can recognize infected or damaged cells and trigger a death pathway through cytotoxic proteins. Under sustained or repeated stimulation, the immune system gradually would lose its normal function, leading to a decrease in immune cell infiltration and the occurrence of immune exhaustion. Hence, we found the cytotoxic T cells in the high-risk group were closer to the tumor cells in this study. Combined with the previous reports, we hypothesize that this phenomenon may facilitate the exhaustion or dysfunction of the infiltrating CD8 + T cells in PC microenvironment by potential mechanisms. The number and function of immune system cells are affected, leading to a decline in immune function, forming a malignant cycle that accelerates tumor progression. The specific mechanism underlying immune exhaustion in high-risk patients is currently unclear, and we will further investigate this phenomenon.

The present study also has some limitations. This study is retrospective, and the prognostic model need to be further validated by prospective data. The mechanistic studies of prognostic model genes are still lacking, which will be added in our next work. Furthermore, single omics data studies are still limited. The multi-omics and integrated analysis for high-throughput data from multiple levels and sources is our further research direction.

## Conclusion

In conclusion, this study was aimed to establish a prognostic risk model for glycosylation-related genes in PC based on the joint TCGA-GTEx database. The GRGs-based prognostic risk model can accurately predict the prognosis, immune microenvironment and immunotherapy efficacy of PC patients. The feasibility and accuracy of the model were validated using pancreatic cancer tissue samples. Glycosylation-related genes may help to predict prognosis and create personalized immunotherapy. We also selected B3GNT8 among the model genes for functional experiments, discovering B3GNT8 may be a promising target for PC therapy. All in all, our model may be a valuable tool for PC risk classification to help clinicians in clinical practice for more personalized treatment and to prolong PC patients’ survival.

### Supplementary Information

**Additional file 1:** **Supplementary Figure 1.** Differential expression of model genes in TCGA GTEx database. **Supplementary Figure 2.** Differential expression of immune cells in high and low risk groups. **Supplementary Figure 3.** Prediction of drug sensitivity in high and low risk groups. **Supplementary Figure 4.** Mutation landscape in risk score. **Supplementary Figure 5.** Performing Kaplan Meier survival analysis using external validation sets from GEO datasets. **Supplementary Figure 6.** B3GNT8 plays a role in inhibiting pancreatic cancer growth and metastasis. **Table S1.** Cell phenotypes and corresponding channel information.

## Data Availability

All data generated or analyzed during this study have been detailed and located in the attachment section of this article, which were available to other researchers following publication.
