# Supplementary material for: Comprehensive prognostic and immune analysis of a glycosylation related risk model in pancreatic cancer
Source: BMC Cancer. 2023 Dec 14;23:1229. doi: 10.1186/s12885-023-11725-1 (PMC10720206; doi:10.1186/s12885-023-11725-1)

Supplementary Data

Supplementary Figure. 1 Differential expression of model genes in TCGA-GTEx database.

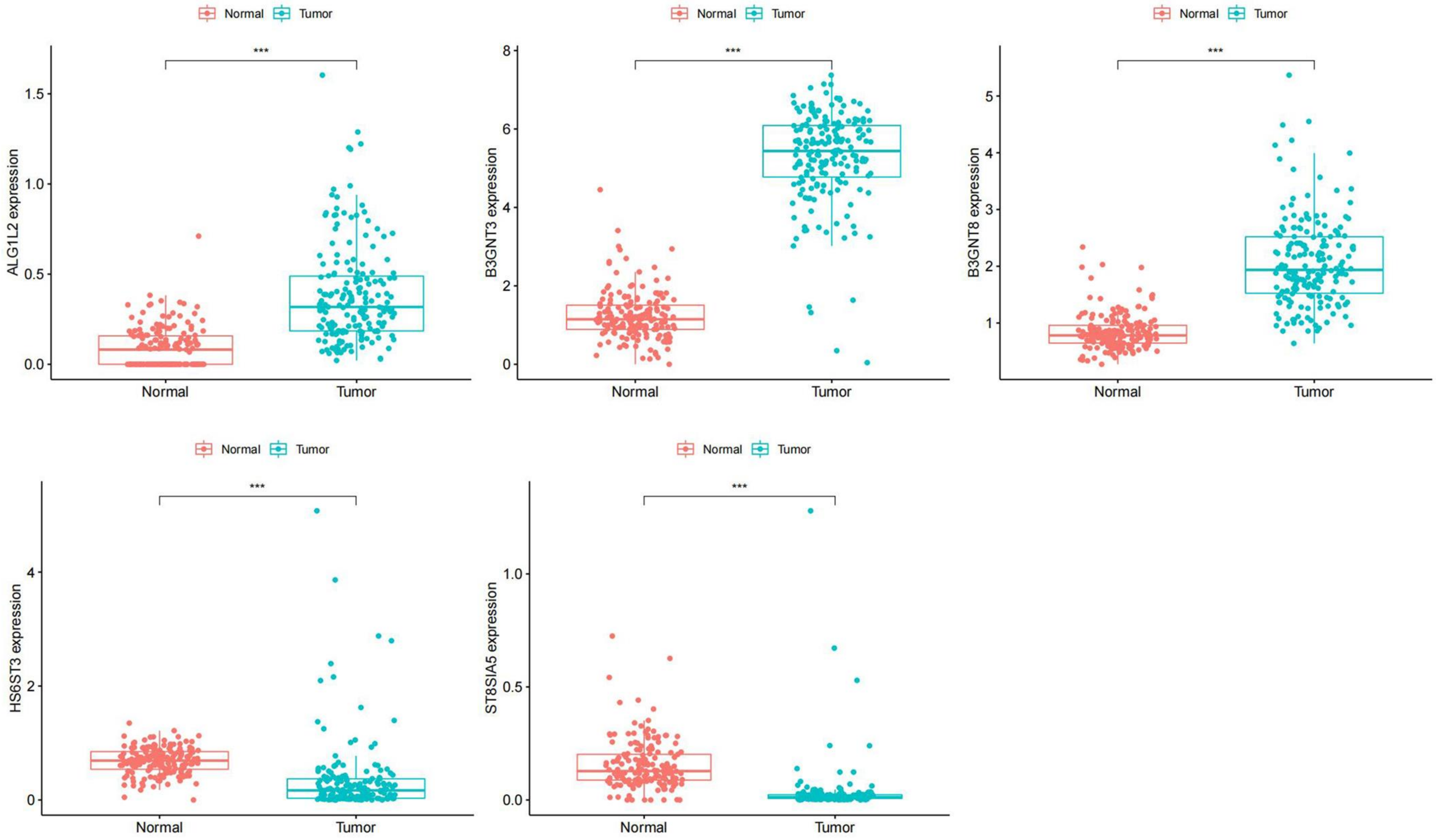

Supplementary Figure.2 Differential expression of immune cells in high and low risk groups.

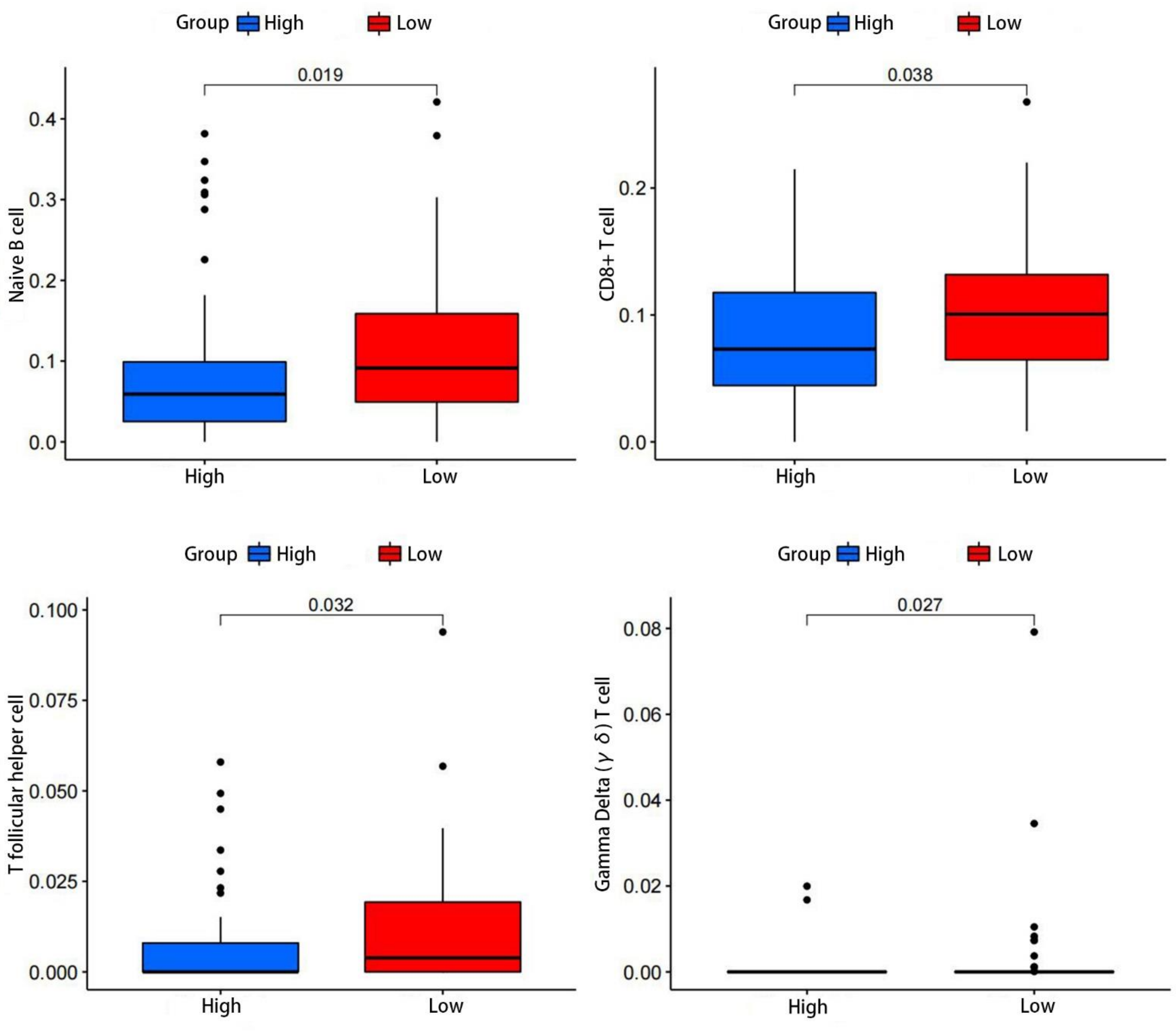

Supplementary Figure.3 Prediction of drug sensitivity in high and low risk groups.

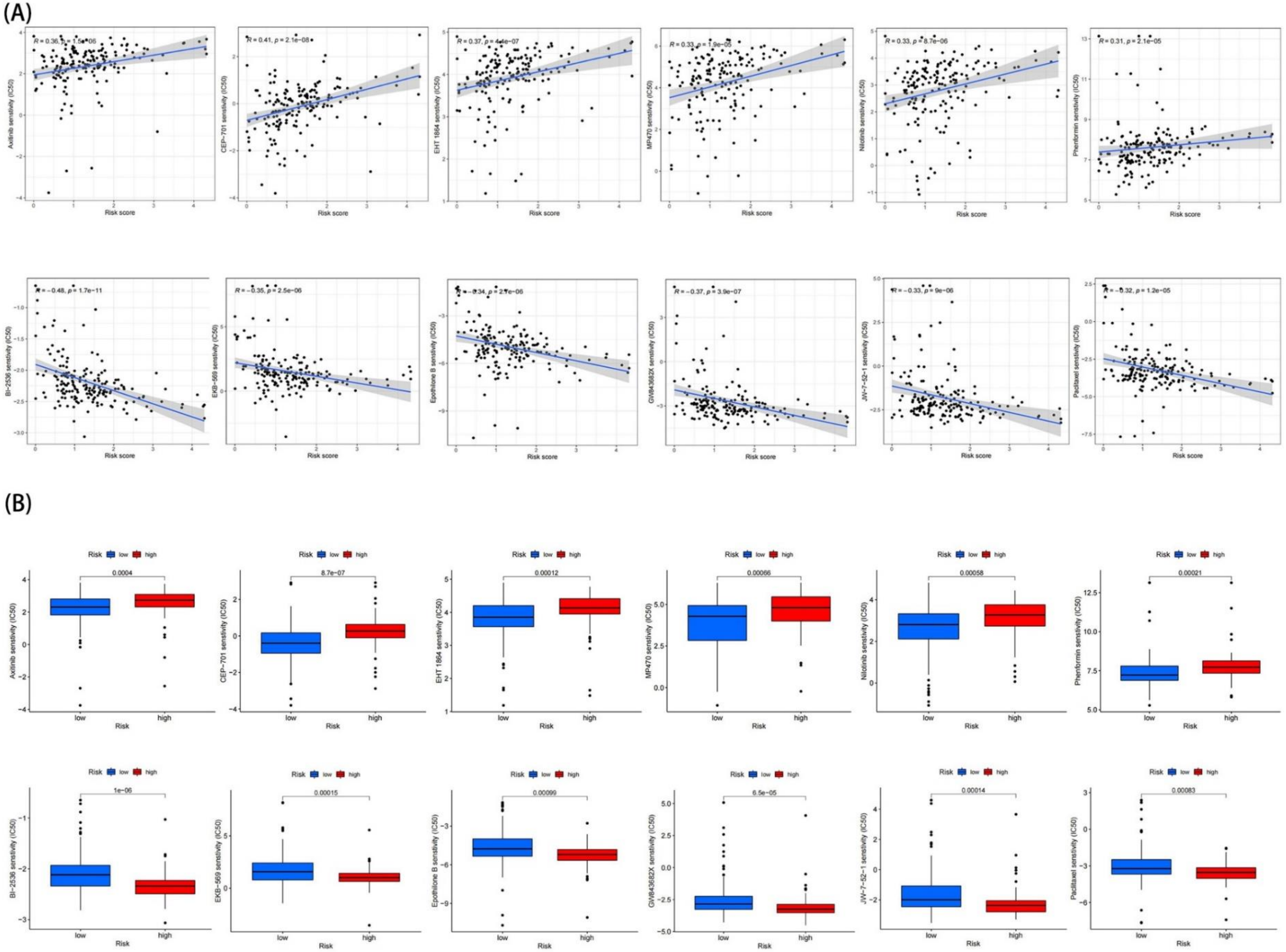

The pRophetic algorithm was used to predict the IC50 and correlation of 35 commonly used chemotherapeutic agents in the high- or low- risk groups.

(A) Risk model was predicted to present a positive correlation with Axitinib, CEP-701, EHT 1864, MP470, Nilotinib and Phenformin, and a negative correlation with Epothilone B, JW-7-52-1, GW843682X, Paclitaxel, BI-2536 and EKB-569.

(B) Predicted IC50 for high and low risk groups.

Supplementary Figure.4 Mutation landscape in risk score.

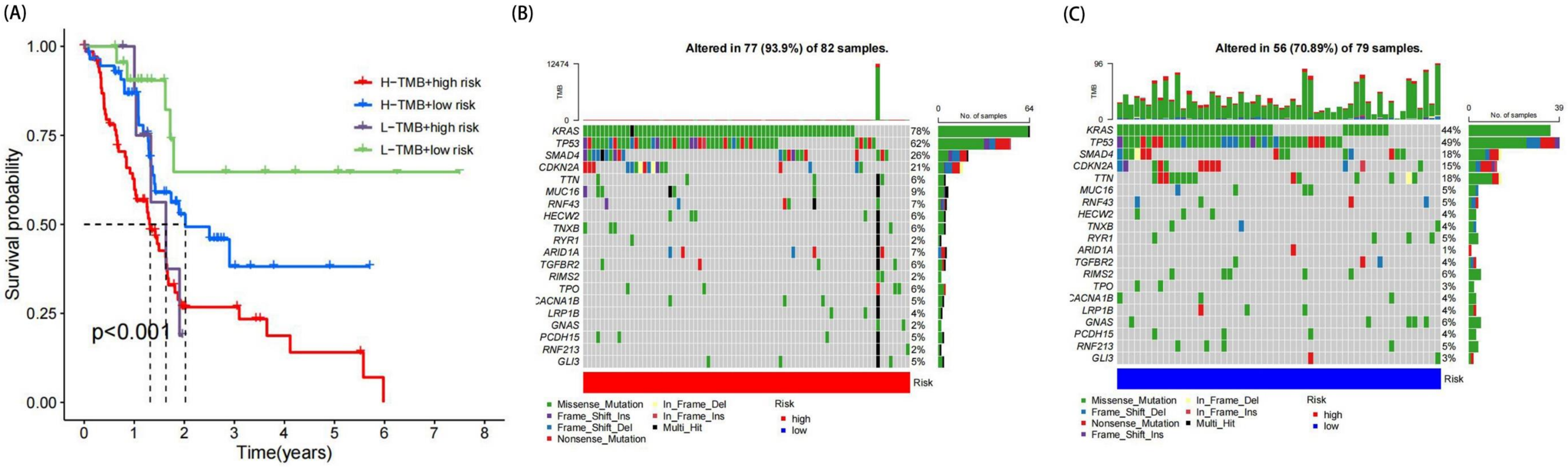

(A) Mutation combined risk score for KM survival analysis.
(B) Plot of the top 20 mutated genes in the high-risk groups.
(C) Plot of the top 20 mutated genes in the low-risk groups. The upper bars show the amount of mutations and the corresponding mutation types. The right bar graph shows the mutation frequencies of the top 20 mutated genes.

Supplementary Figure.5 Performing Kaplan-Meier survival analysis using external validation sets from GEO datasets.

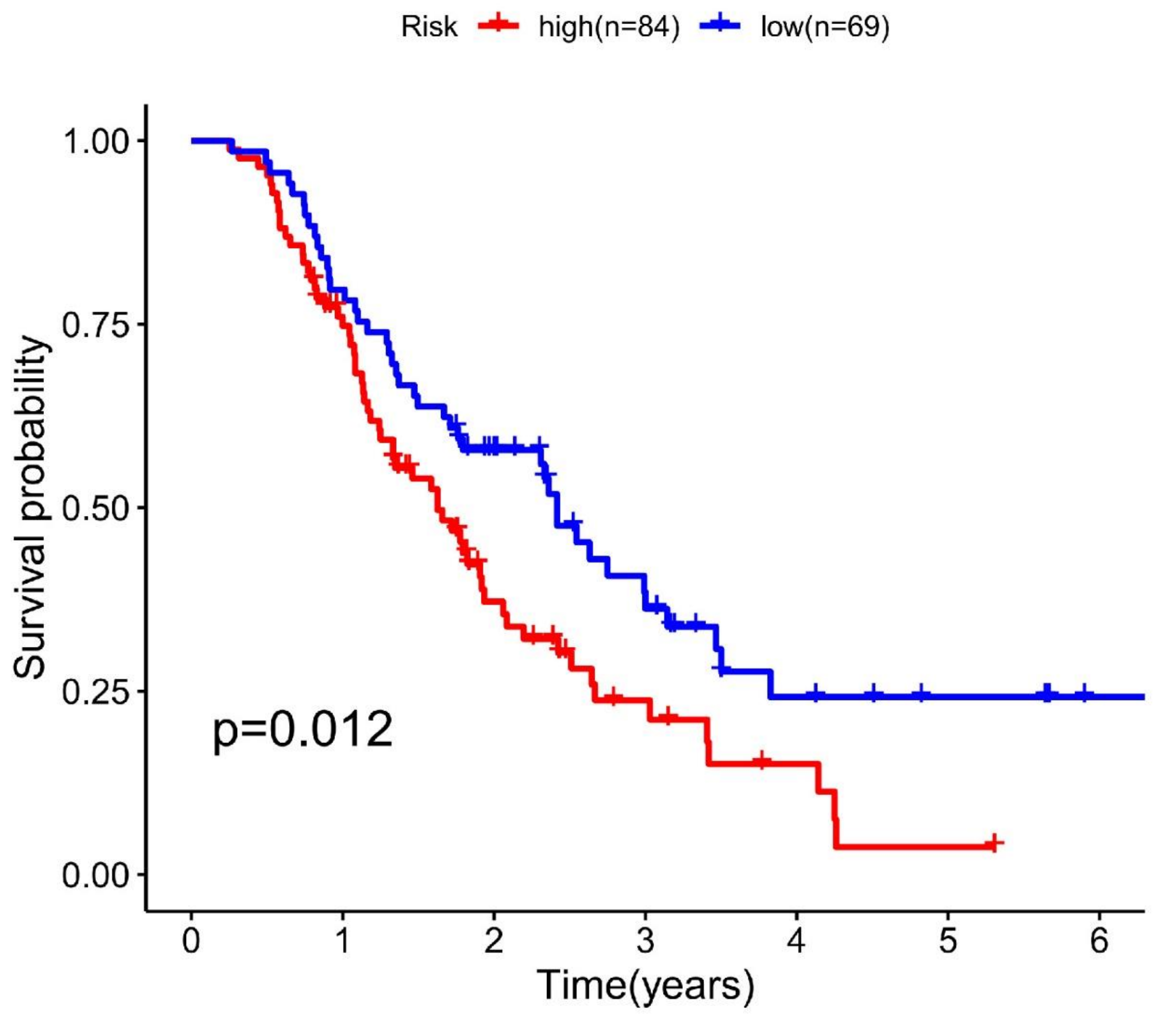

Supplementary Figure.6 B3GNT8 plays a role in inhibiting pancreatic cancer growth and metastasis.

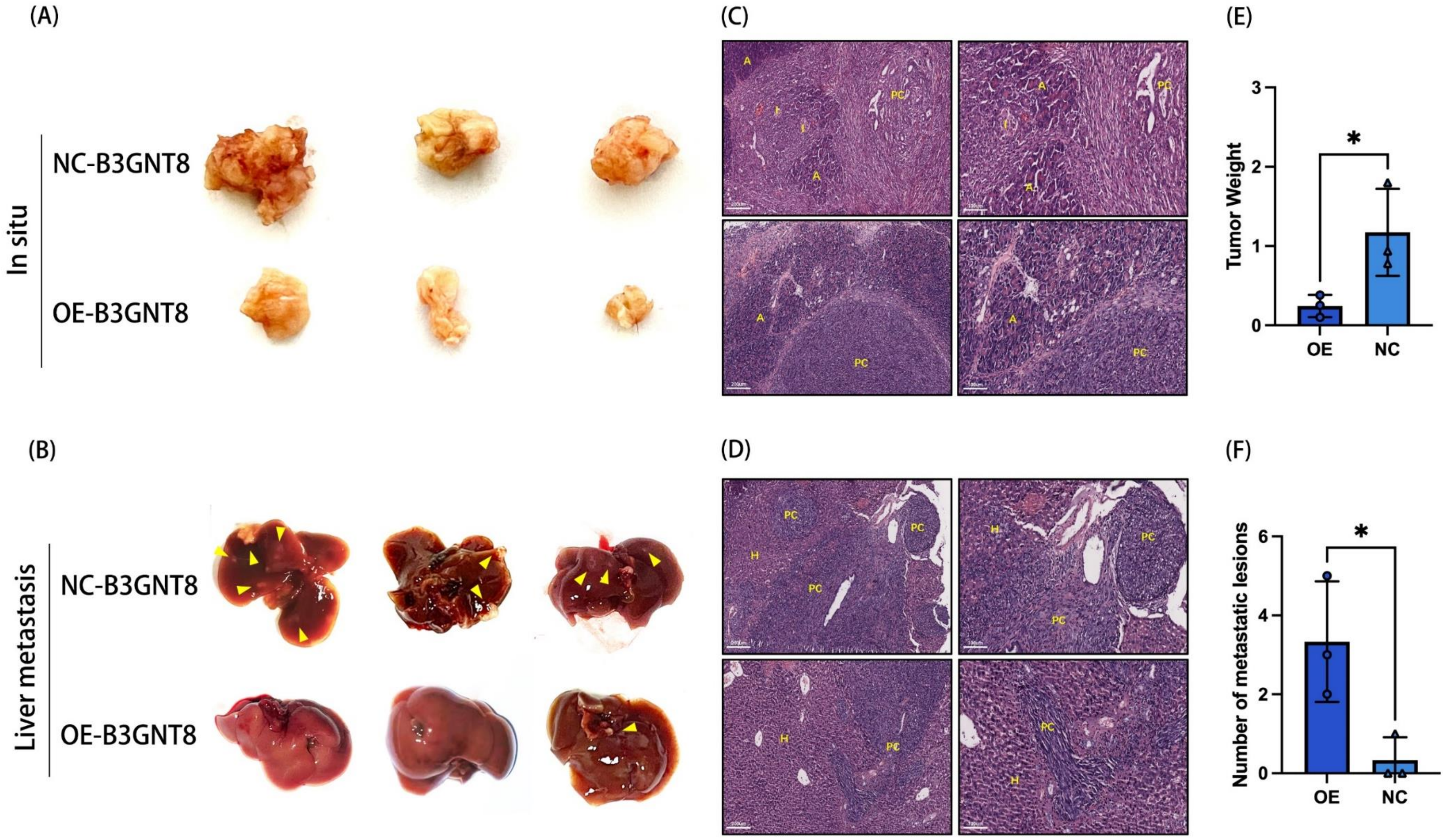

(A) In situ tumor formation in different B3GNT8 expression groups.
(B) Liver metastasis in different B3GNT8 expression groups
(C) HE staining images at 10x magnification and 20x magnification of in situ tumors from different B3GNT8 expression groups (A is for pancreatic alveoli, I is for pancreatic islets, PC is for pancreatic cancer).
(D) HE staining images at 10x magnification and 20x magnification of liver metastases from different B3GNT8 expression groups (PC is for pancreatic cancer, H is for Hepatocyte).
(E) Differential statistics of tumor weight in different B3GNT8 expression groups.
(F) Differential statistics of the number of transfer lesions in different B3GNT8 expression groups.

Table S1 Cell phenotypes and corresponding channel information.

Table.1: cell phenotypes and corresponding channel information

Index	Phenotype Name	Dye Name	type1	type2
0	PANCK+	CY5	AND	Positive
1	PDL1+	SpGold	AND	Positive
2	CD8+	SpGreen	AND	Positive
3	PDL1+CD8+	SpGold	AND	Positive
3	PDL1+CD8+	SpGreen	AND	Positive
4	PDL1+PANCK+	SpGold	AND	Positive

Note: Index: Cell Phenotype ID; Phenotype Name: Cell Phenotype Name; Dye Name: Channel Name in the scan results; type1: Relationship between channels within a single phenotype. If the relationship between channels within a phenotype is "and," it means all channels must be present; if the relationship is "or," it means any of the channels can be present. type2: If the channel is positive within a single phenotype, it is labeled as Positive; if it is negative, it is labeled as Negative.

Original images

Original images of Western Blots

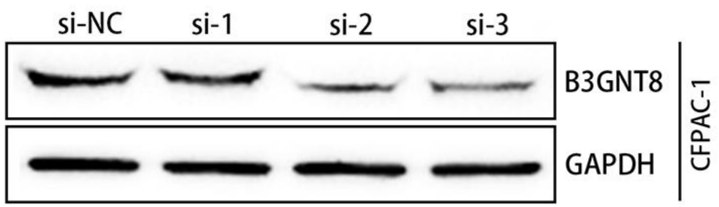

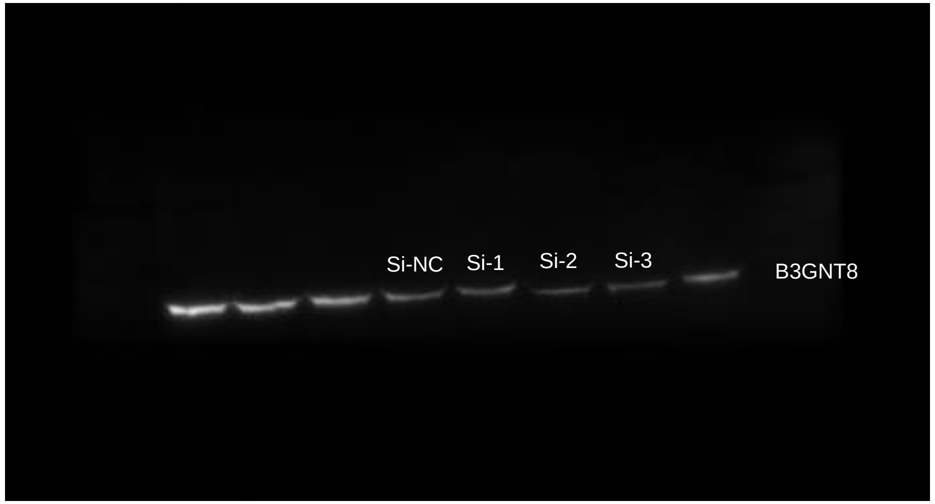

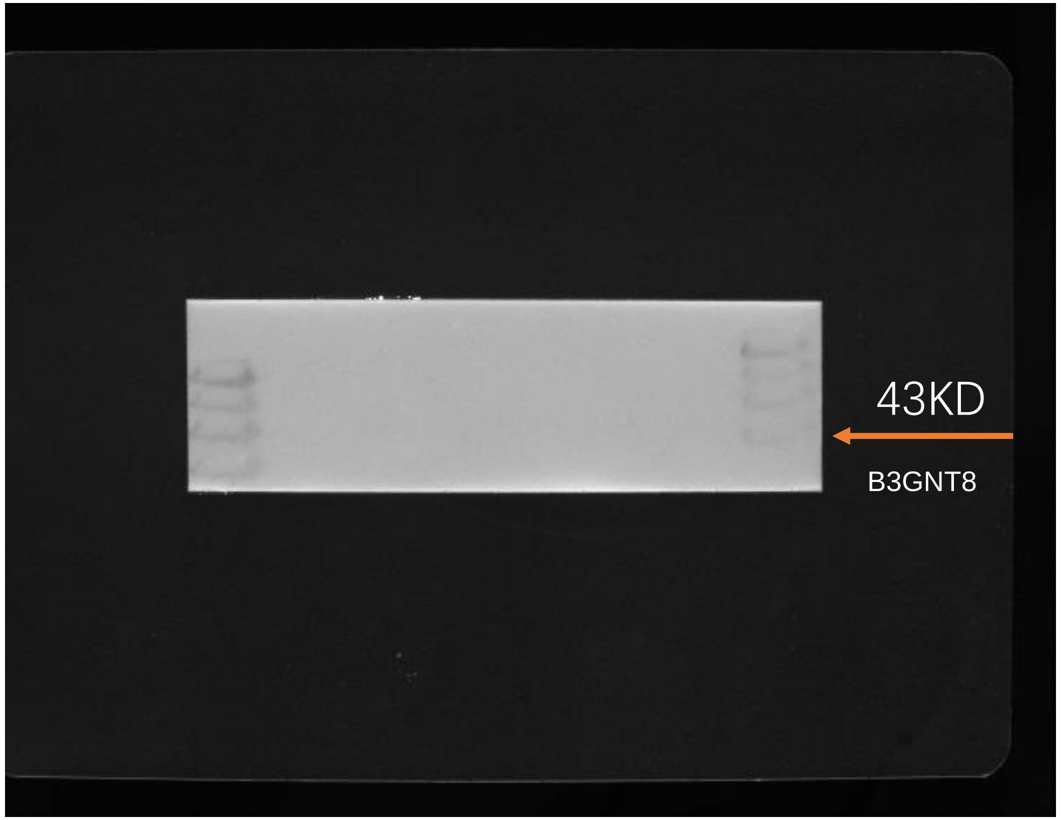

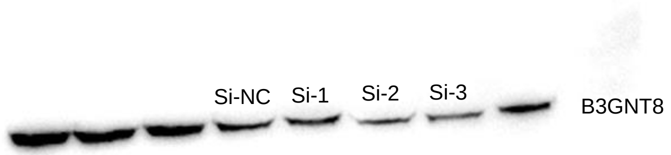

Original images of Western Blots

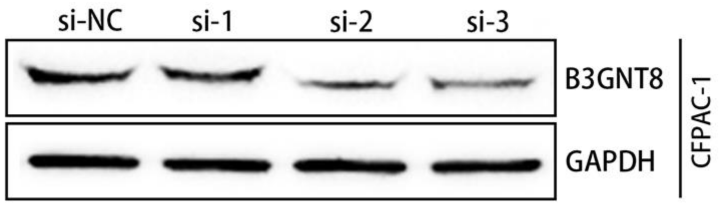

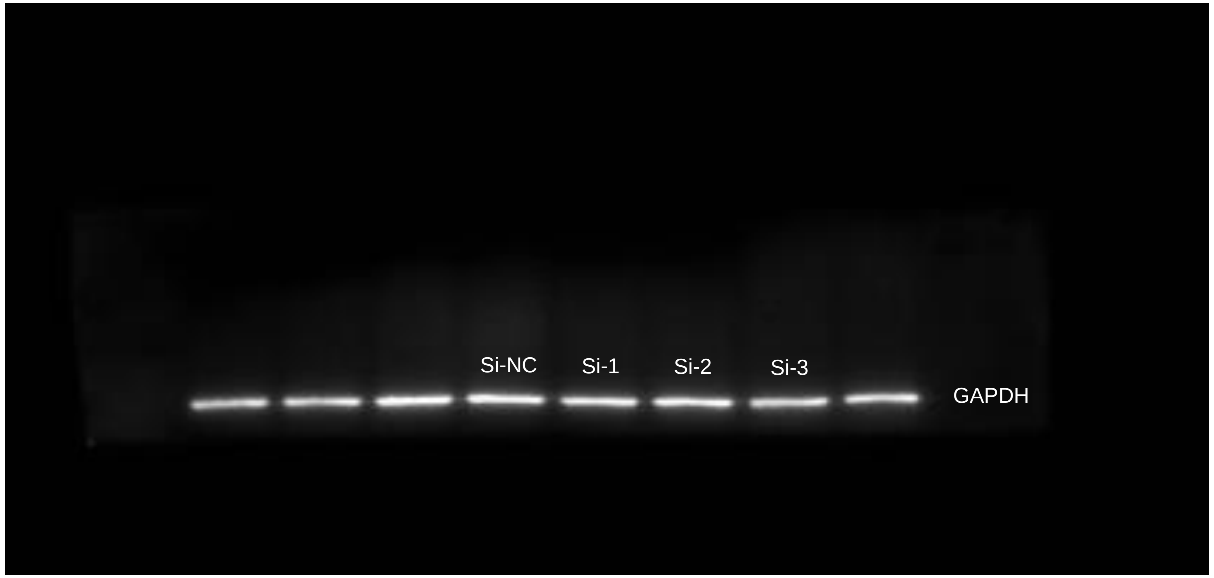

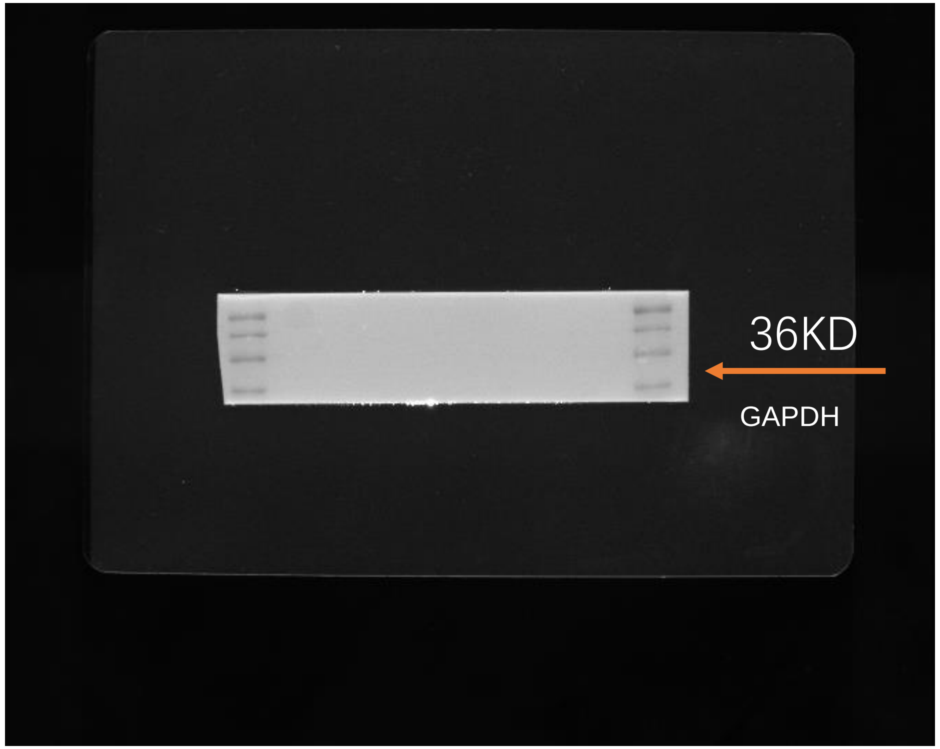

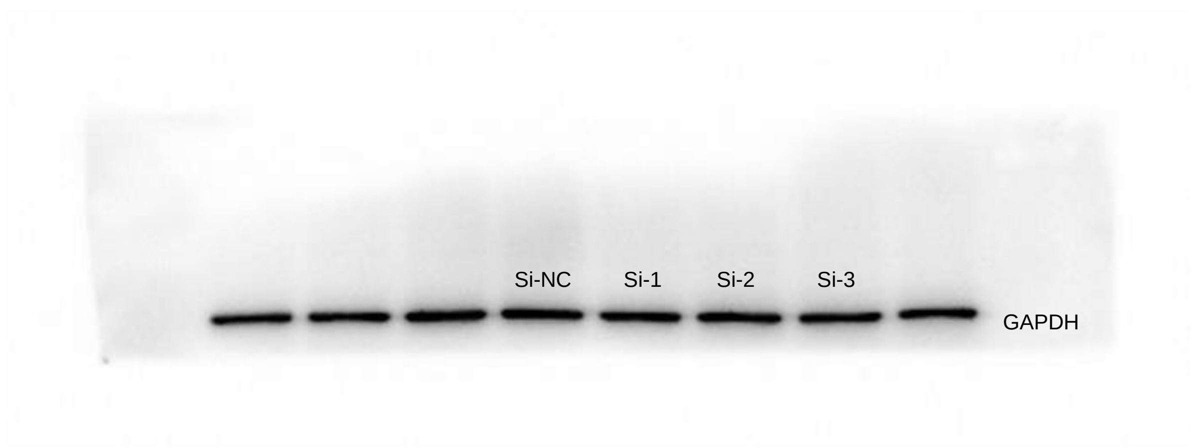

Original images of Western Blots -- **Replication**

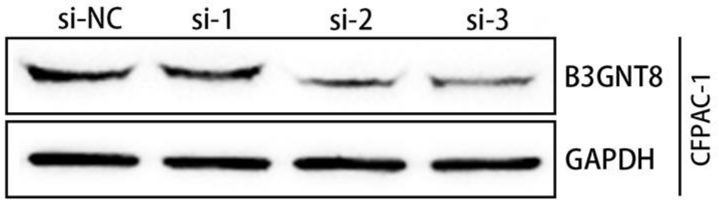

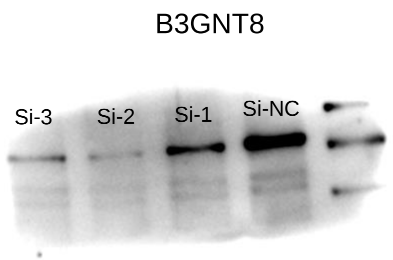

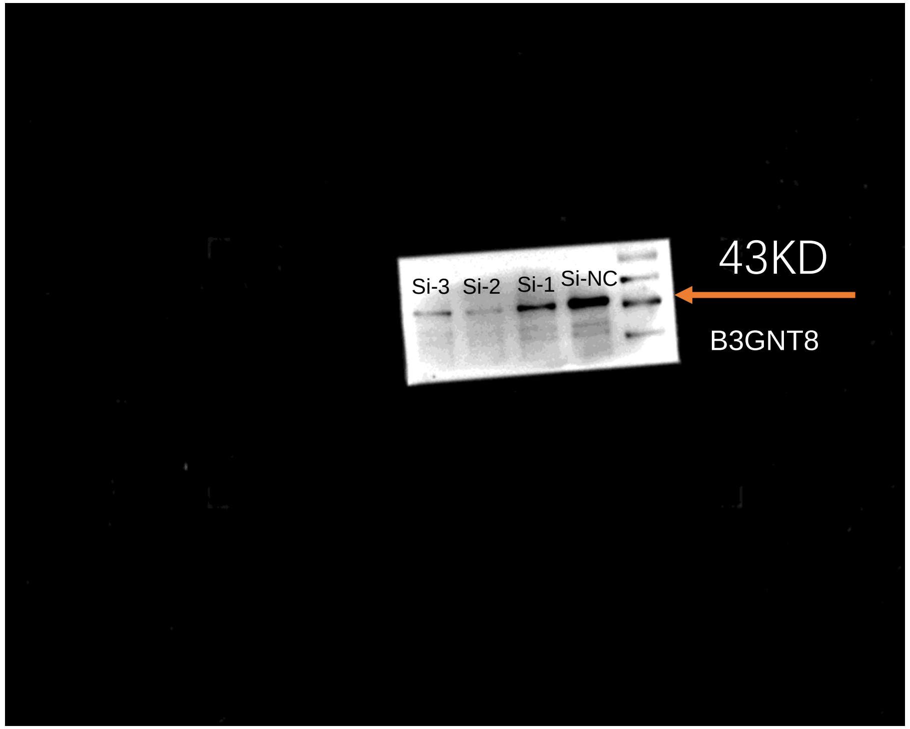

Original images of Western Blots -- **Replication**

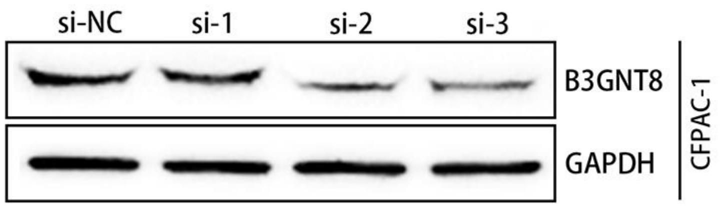

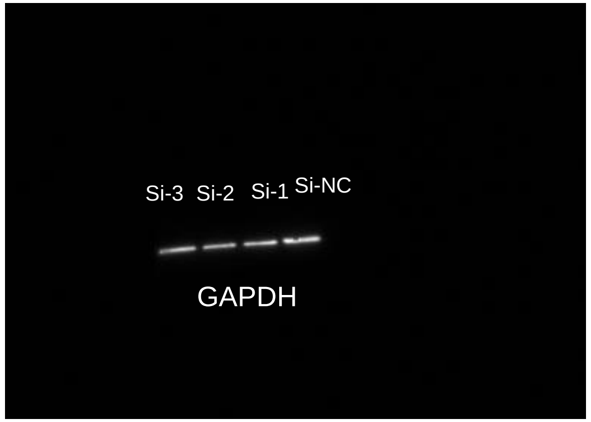

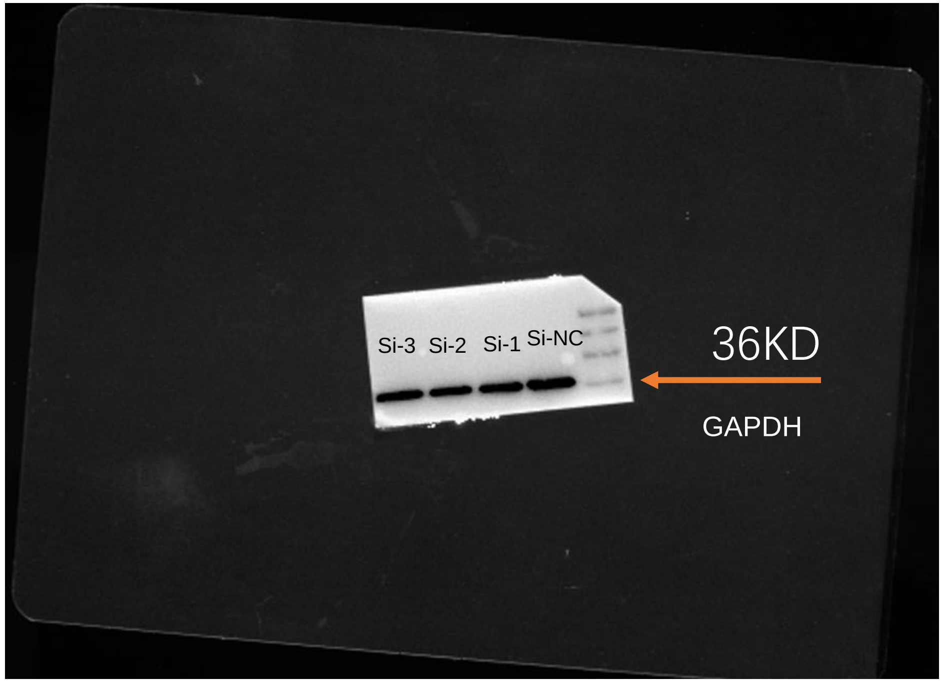

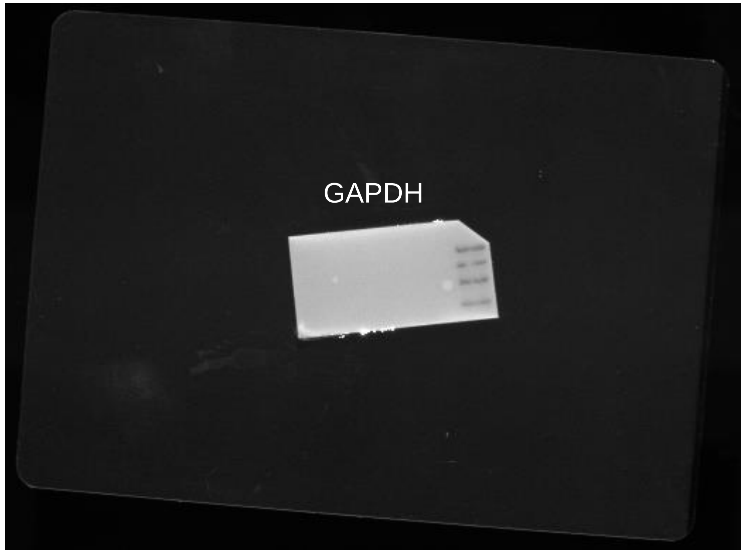

Original images of Western Blots

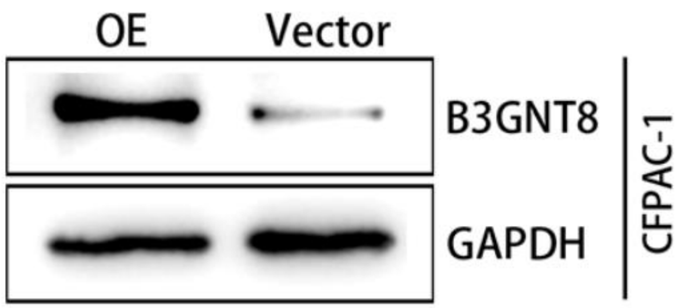

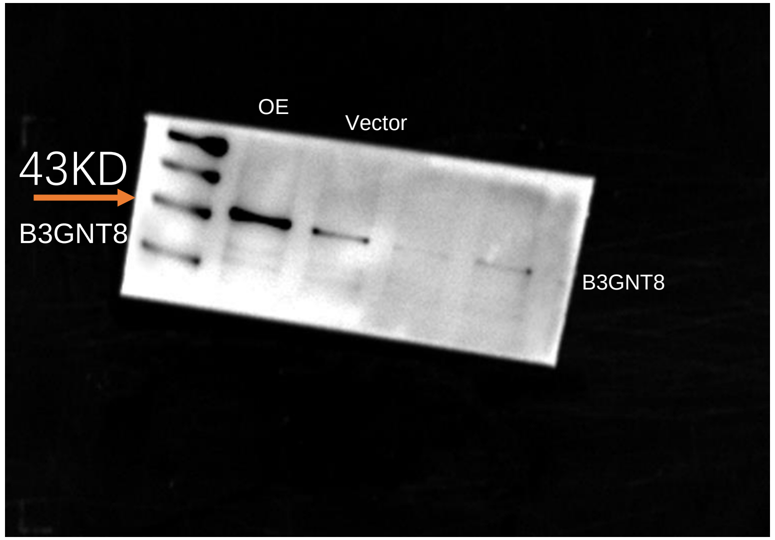

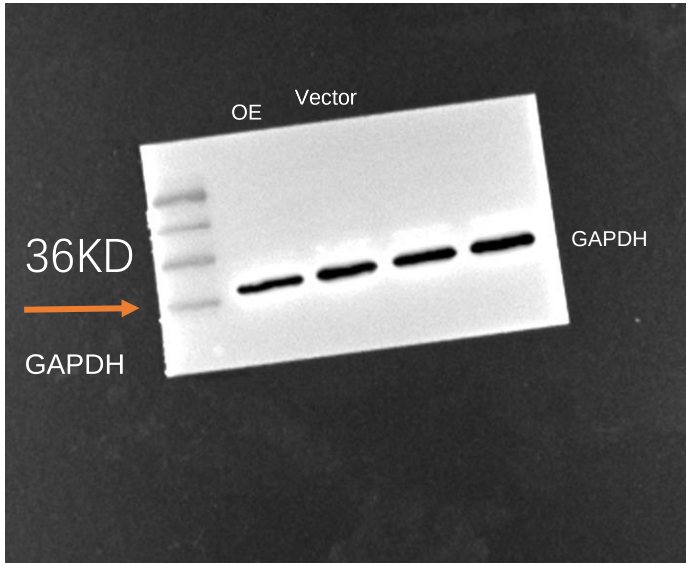

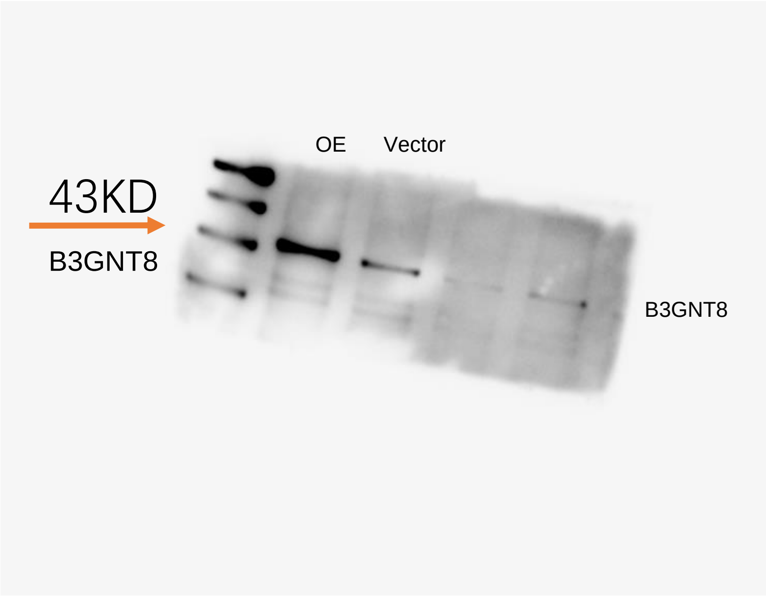

Original images of Western Blots -- **Replication**

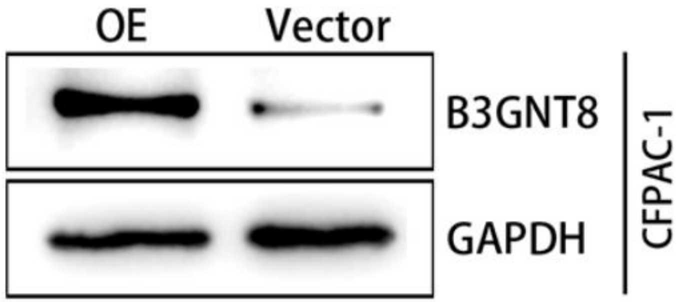

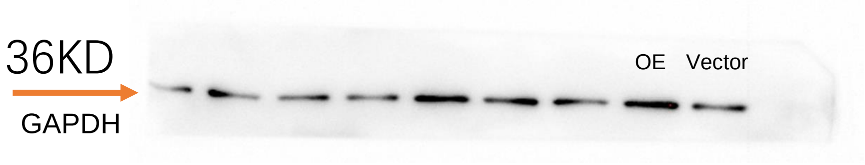

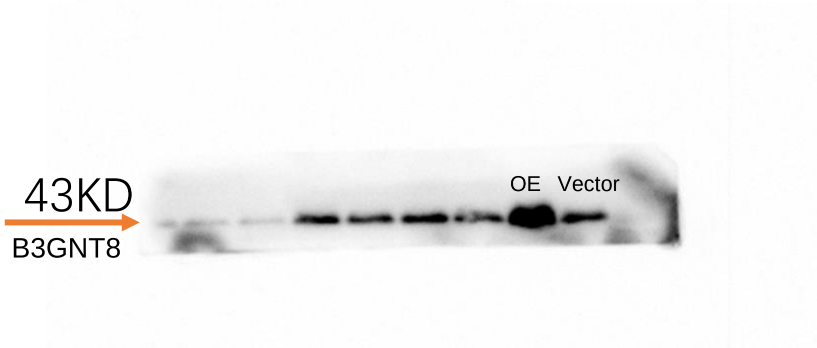

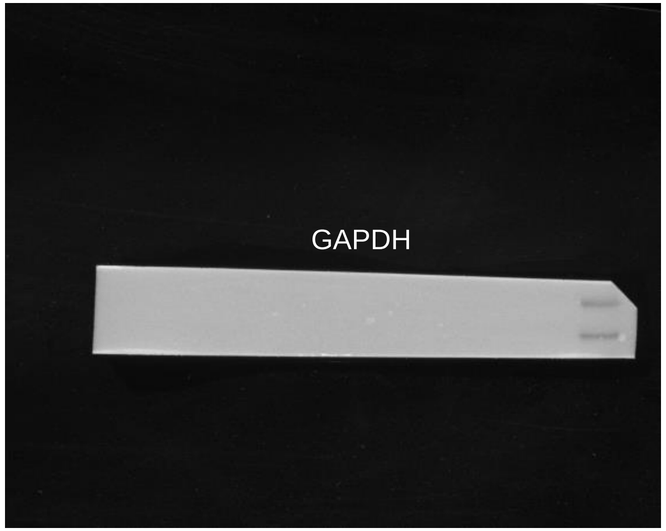

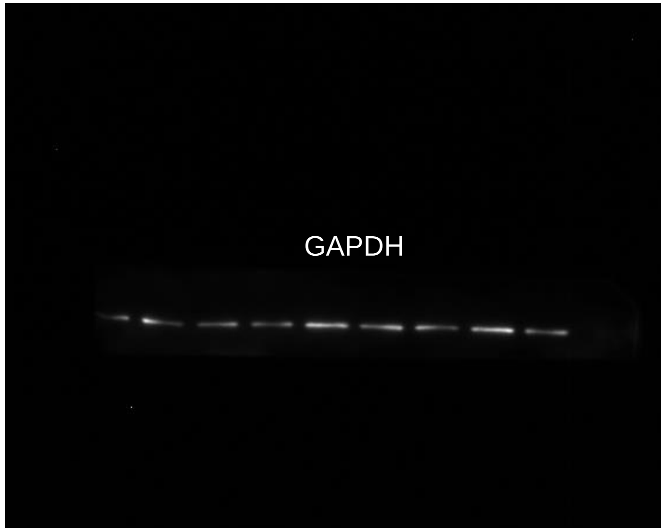

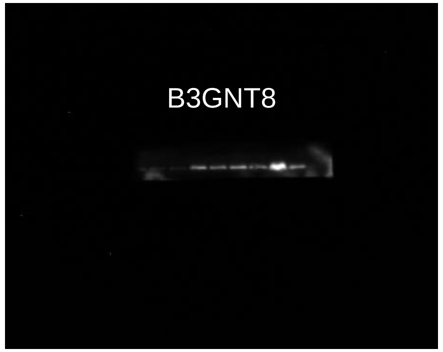

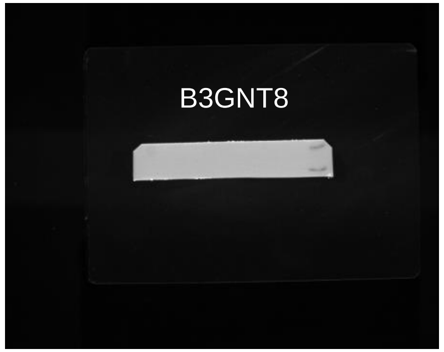

Original images of Western Blots

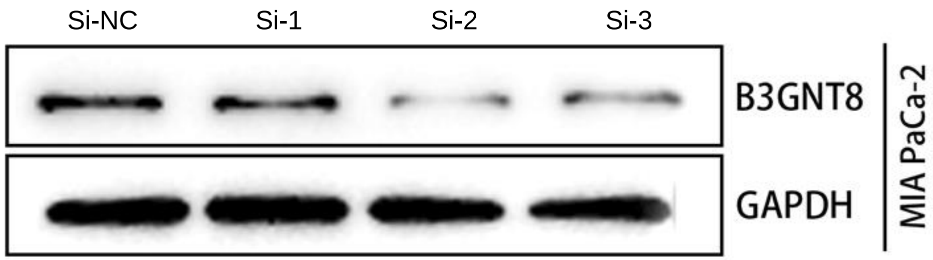

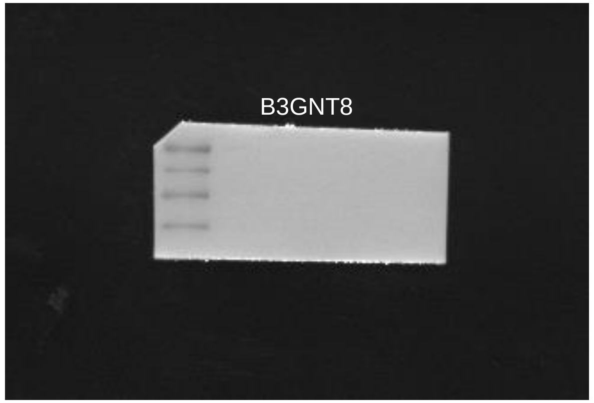

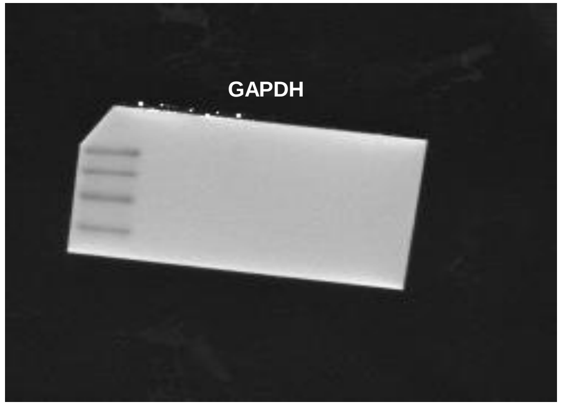

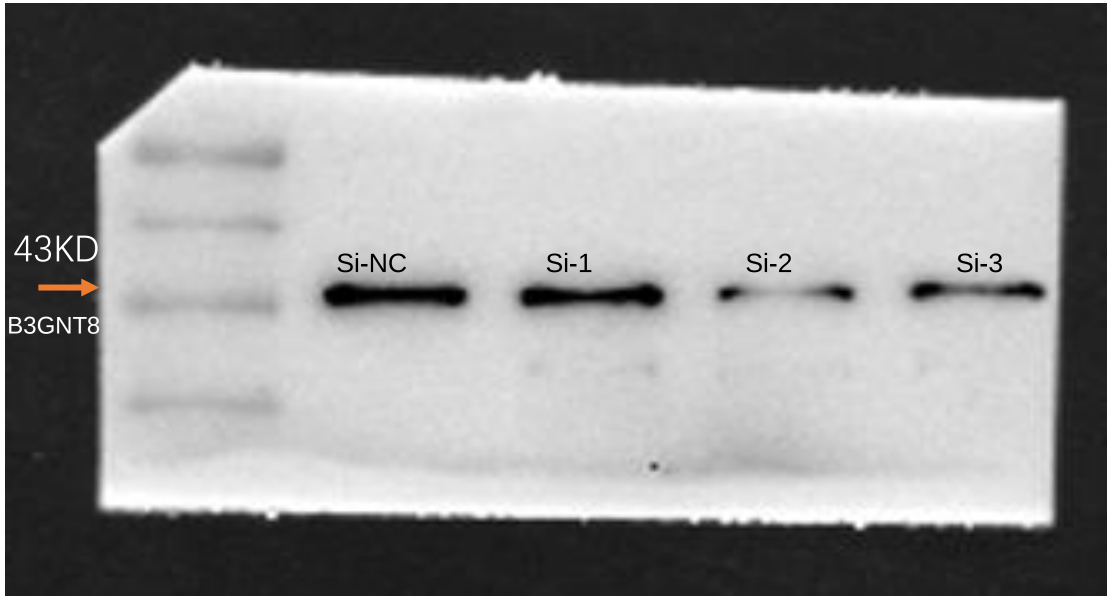

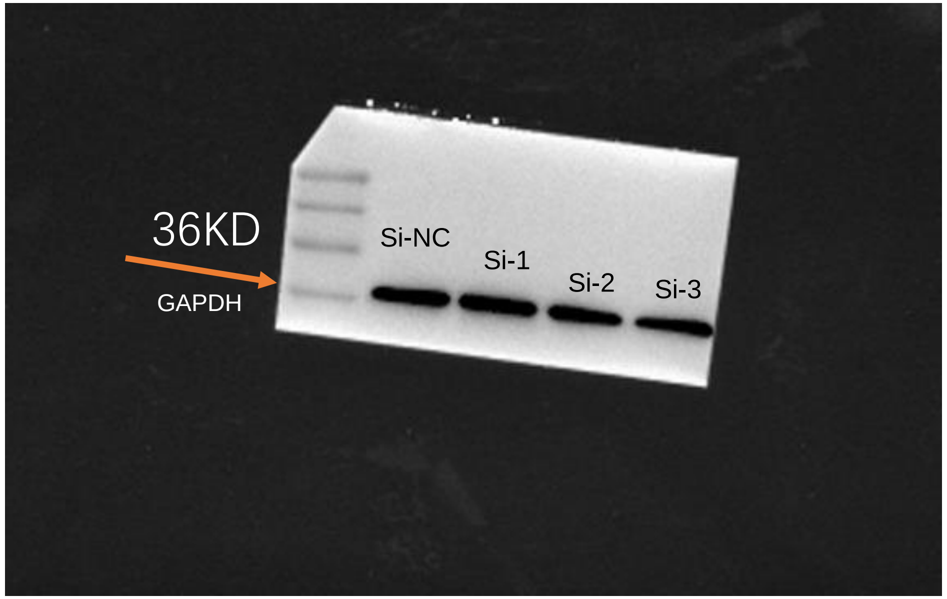

Original images of Western Blots -- **Replication**

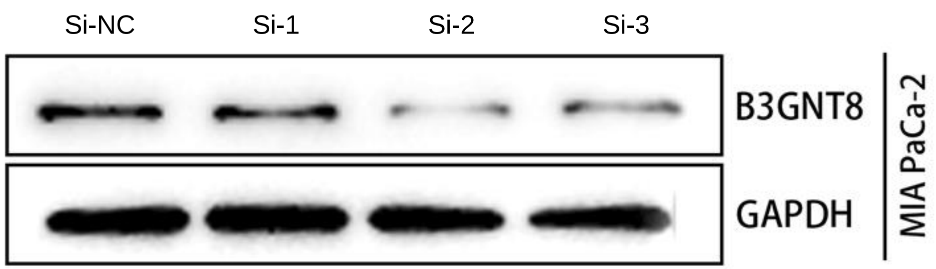

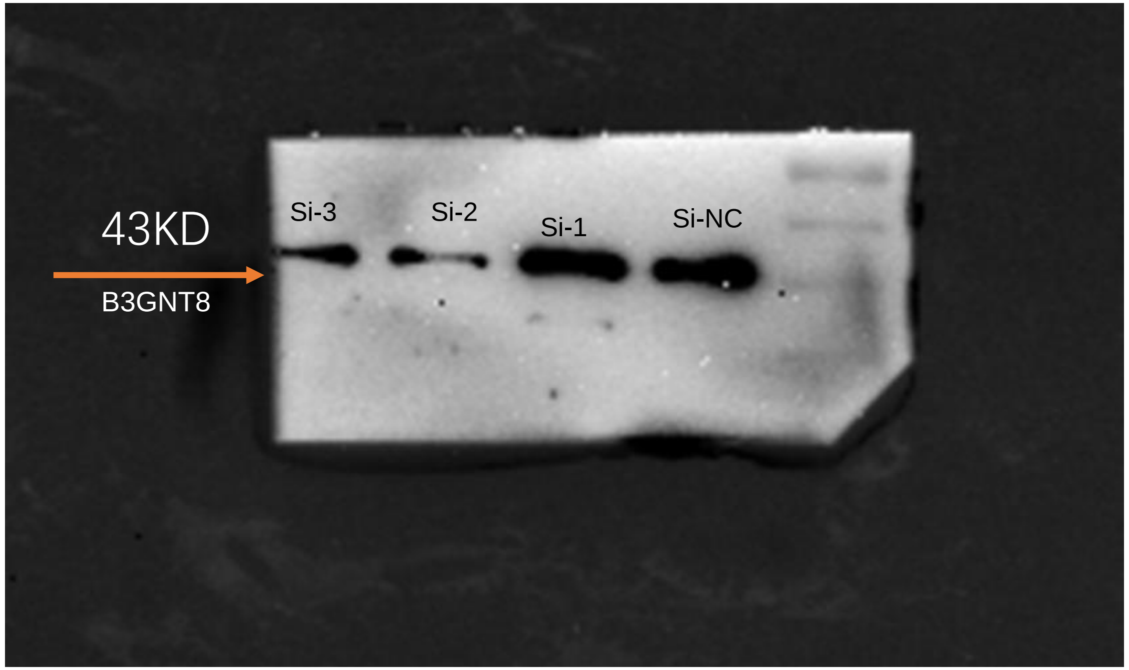

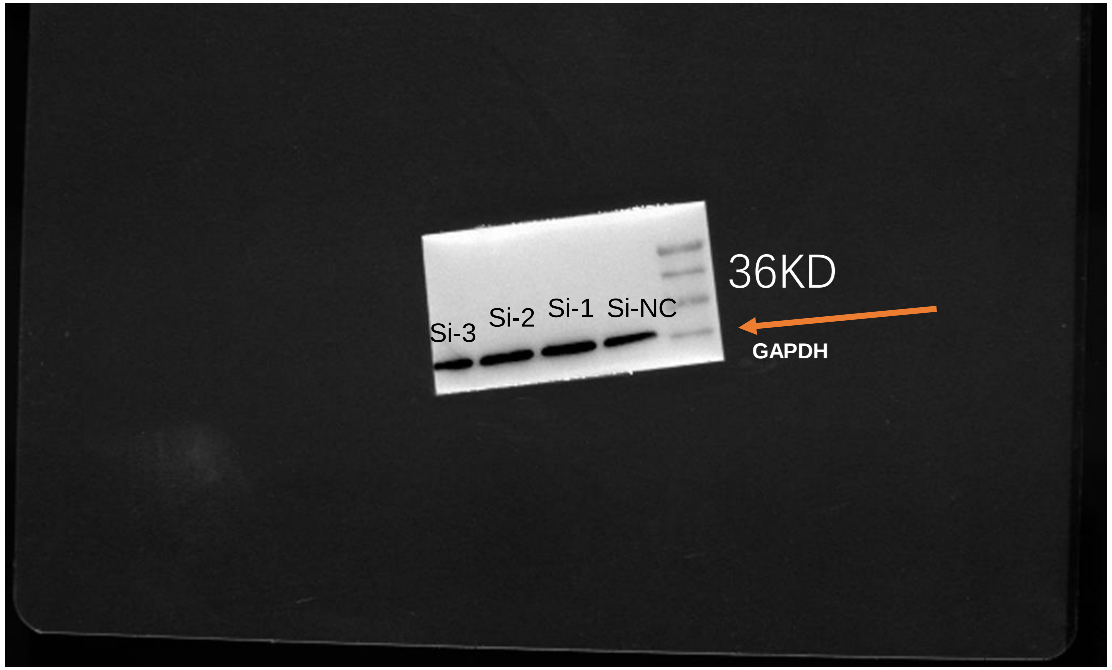

Original images of Western Blots

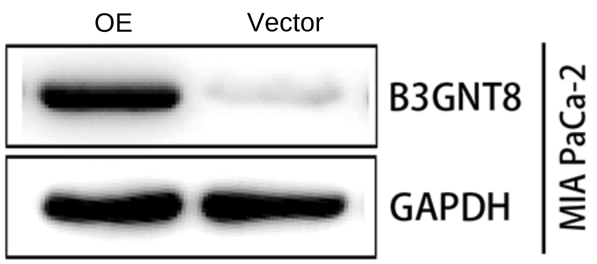

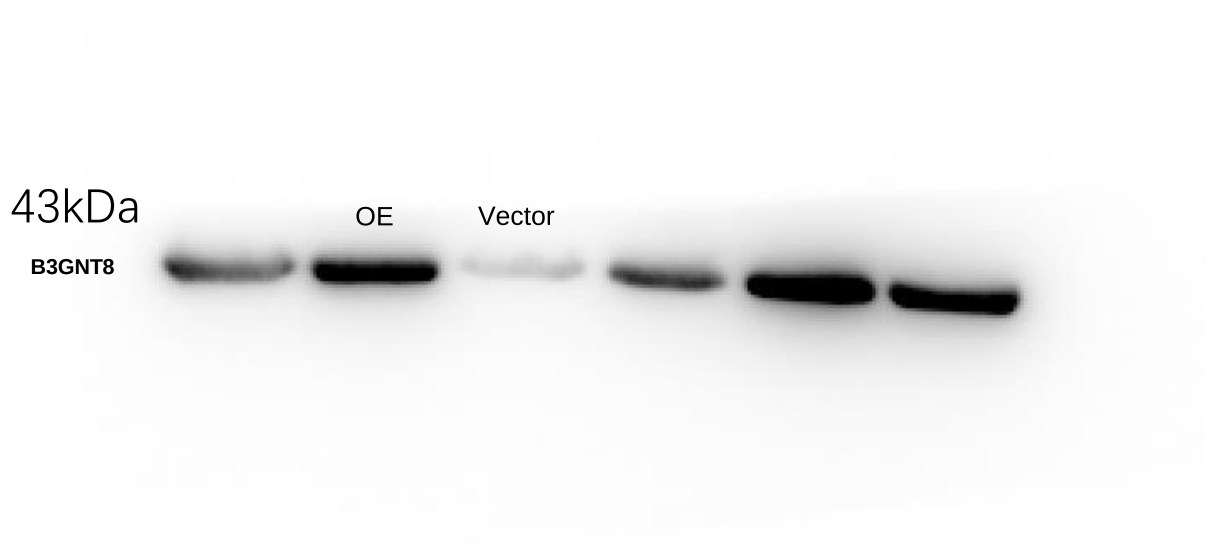

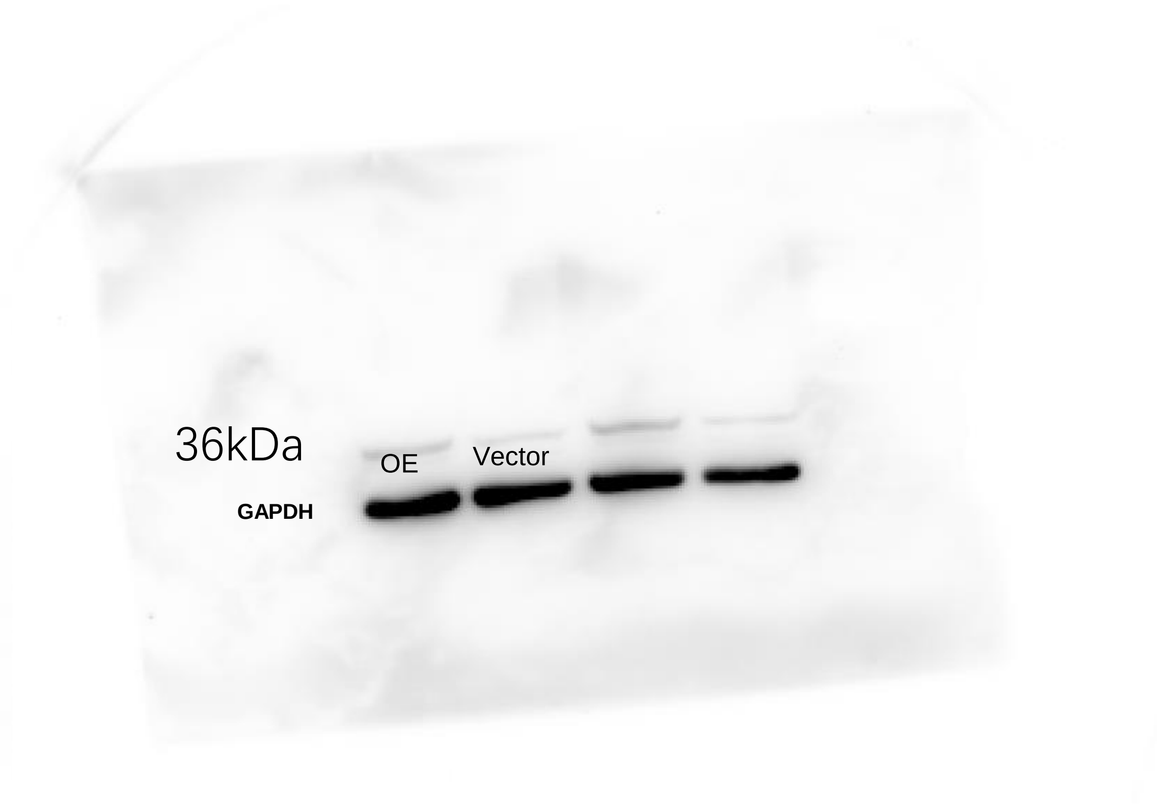

Original images of Western Blots -- **Replication**

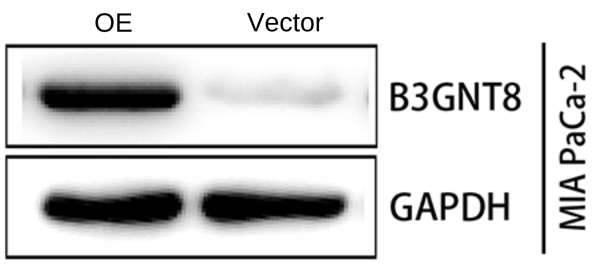

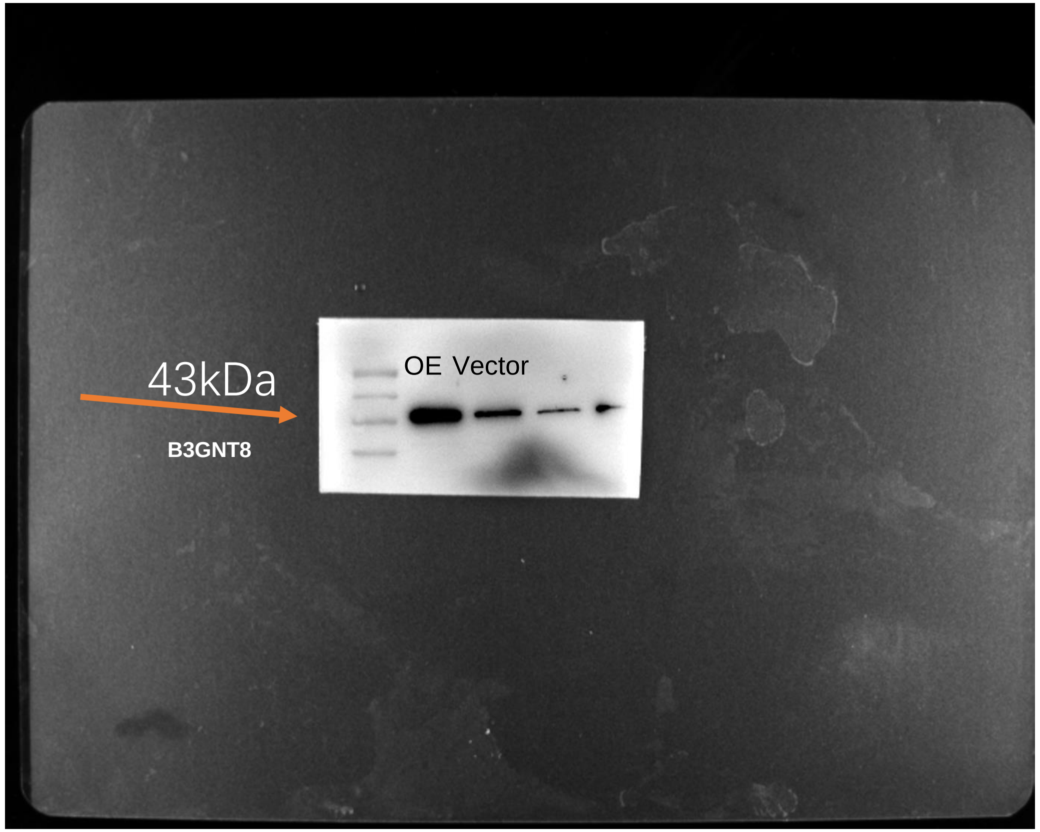

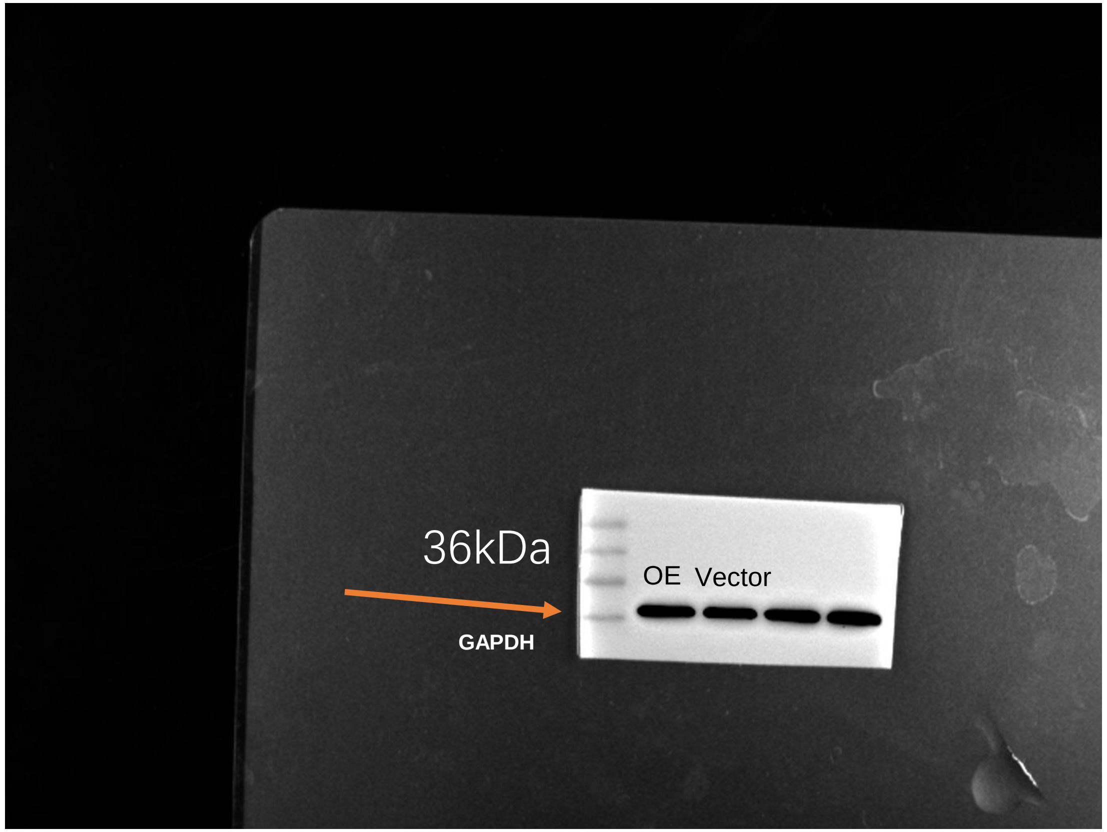

Supplement: Supplementary file 1 — Additional file 1: Supplementary Figure 1. Differential expression of model genes in TCGA GTEx database. Supplementary Figure 2. Differential expression of immune cells in high and low risk groups. Supplementary Figure 3. Prediction of drug sensitivity in high and low risk groups. Supplementary Figure 4. Mutation landscape in risk score. Supplementary Figure 5. Performing Kaplan Meier survival analysis using external validation sets from GEO datasets. Supplementary Figure 6. B3GNT8 plays a role in inhibiting pancreatic cancer growth and metastasis. Table S1. Cell phenotypes and corresponding channel information. [file 12885_2023_11725_MOESM1_ESM.pdf]
